# Benchmarking DNA barcode decoding strategies under high error rates

**DOI:** 10.1186/s12859-026-06540-x

**Published:** 2026-06-23

**Authors:** Franco Poma-Soto, Hanne Van Droogenbroeck, Brecht Soulliaert, Maya Giridhar, Jürgen Behr, Hamed Sabzalipoor, Mark Somoza, Pieter Mestdagh, Jo Vandesompele

**Affiliations:** 1 Department of Biomolecular Medicine, Ghent University, Corneel Heymanslaan 10, 9000 Ghent, Belgium; 2 OncoRNALab, Cancer Research Institute Ghent (CRIG), Ghent University, Corneel Heymanslaan 10, 9000 Ghent, Belgium; 3 Leibniz Institute for Food Systems Biology at the Technical University of Munich, Technical University of Munich, Lise-Meitner-Straße 30, 85354 Freising, Germany; 4 Chair of Food Chemistry and Molecular Sensory Science, Technical University of Munich, Lise-Meitner-Straße 34, 85354 Freising, Germany; 5 Institute of Inorganic Chemistry, Faculty of Chemistry, University of Vienna, Josef-Holaubek-Platz 2, 1090 Vienna, Austria

**Keywords:** Barcode calling, High-throughput sequencing, Benchmarking, Error correction, Demultiplexing, Photolithography, Spatial transcriptomics

## Abstract

**Background:**

DNA barcoding enables multiplexed identification of biomolecules in pooled sequencing experiments, with broad applications including spatial transcriptomics. Photolithographic synthesis of high-density barcode arrays achieves library sizes exceeding $10^5$ unique sequences but introduces error rates of 10–20% per nucleotide through substitutions, insertions, and deletions. Classical error-correcting codes cannot scale to such library sizes while maintaining robust error correction under these conditions.

**Methods:**

We benchmarked three computational barcode decoding approaches—Columba (FM-index-based lossless alignment), QUIK (k-mer filtering with GPU acceleration), and RandomBarcodes (trimer-based triage with GPU parallelization)—across simulated and empirical datasets. Simulations spanned barcode lengths of 28–36 nt, library sizes of 21,000–85,000 barcodes, and error rates of 9–32%. Real sequencing data were generated from photolithographically synthesized arrays at three printing density levels.

**Results:**

Under medium error rates (~23%), QUIK achieved the highest recall (87–89%) while maintaining precision *>99.5%*, outperforming RandomBarcodes (recall 56%, precision *>99.8%*) and Columba (recall 35%, precision 98–100%). QUIK demonstrated superior scalability, processing 59,620 reads/second on a single GPU compared to RandomBarcodes (68 reads/second) and Columba (1550 reads/second with 8 CPU threads). Barcode length strongly influenced accuracy: 34-nt barcodes enabled 75% recall at 99.97% precision with QUIK, compared to 60% recall with 32-nt barcodes. On real data from a 42,000-spot subarray with 36-nt barcodes, QUIK managed a 57% assignment rate with perfect precision, versus 52% (Columba, precision 99.96) and 50% (RandomBarcodes, precision 99.82).

**Conclusions:**

QUIK provides the optimal balance of speed, accuracy, and scalability for high-density spatial transcriptomics applications under realistic synthesis error conditions. Barcode lengths *≥ 34* nt are recommended for applications requiring *>75%* read recovery at *>99.9%* precision.

**Supplementary Information:**

The online version contains supplementary material available at https://doi.org/10.1186/s12859-026-06540-x.

## Background

Predefined sets of short DNA sequences, commonly referred to as DNA barcodes, are widely used in high-throughput sequencing experiments to uniquely label and track individual biomolecules or samples within pooled populations [1, 2]. By attaching a specific barcode to each molecule or sample, sequencing reads generated from a mixed pool can later be assigned to their origin through a process known as barcode calling—a critical step in downstream data analysis. DNA barcoding strategies have enabled a wide range of applications across genomics, from multiplexed library preparation to single-cell profiling [3, 4]. A particularly prominent recent application is spatial transcriptomics, which leverages spatially barcoded DNA probes immobilized on solid surfaces to capture transcripts from tissue sections [5, 6], thereby allowing gene expression patterns to be mapped back to their precise spatial locations. In one widely used implementation, high-density oligonucleotide arrays are fabricated via photolithography, producing tens of thousands of unique DNA barcodes at known physical locations on a microscope glass slide [7–9]. Each barcode acts as a spatial identifier, allowing transcripts captured at that spot to be mapped back to their tissue position after sequencing. A fundamental challenge in such systems is the high error rate associated with in situ barcode synthesis and subsequent sequencing. While conventional next-generation sequencing typically exhibits per-base error rates below 1%, photolithographically synthesized barcodes can accumulate substitution, deletion, and insertion errors at rates exceeding 10–20% per base [7, 9]. In one microarray synthesis study, fewer than 0.1% of oligonucleotides were completely error-free in the final library. These synthesis errors are particularly problematic because they are systematic: all reads derived from a given barcode inherit the same underlying errors, greatly complicating barcode recovery. Classical approaches to barcode robustness rely on error-correcting code (ECC) designs that enforce a minimum Hamming or Levenshtein distance between barcode sequences [10, 11]. In principle, such designs guarantee correction of a bounded number of errors. In practice, however, constructing very large barcode libraries with sufficient error-correction capacity is difficult, especially when indels are prevalent [12]. Most ECC-based barcode sets support only modest library sizes (typically $\le 10^4$ barcodes) and assume predominantly substitution errors, limiting their applicability in high-error, indel-rich settings [13]. To address these limitations, several computational decoding strategies have been developed that aim to recover barcode identities from noisy reads without relying on rigid ECC constraints. These methods differ substantially in algorithmic philosophy:

- Alignment-based approaches directly compute edit distances between reads and reference barcodes but scale poorly without aggressive pruning.
- Index-based methods, such as those leveraging FM-indices, accelerate approximate matching by exploiting precomputed searchable structures, enabling lossless alignment at scale [14].
- Filtering-based approaches employ inexpensive heuristics, such as k-mer similarity, to discard implausible candidates before applying more expensive verification steps [13, 15].
- Clustering-based methods infer barcode identities by grouping similar reads and extracting consensus sequences, although their effectiveness is limited when error rates are high and error-free reads are rare [16–18].

Three recent tools exemplify distinct decoding paradigms under high error rates:

- Columba [14] implements optimal search schemes over a bidirectional FM-index to perform lossless approximate pattern matching. While Columba was originally designed as a general-purpose read aligner for applications such as variant calling and metagenomics—where reporting all optimal alignments is essential—its lossless alignment capabilities make it applicable to barcode decoding. We include Columba in this benchmark as a representative of index-based exact alignment methods, recognizing that barcode calling is not its primary intended use case but represents a plausible application of its core functionality.
- RandomBarcodes [13] uses GPU-accelerated k-mer–based filtering followed by edit-distance verification, enabling decoding at error rates up to ~20%.
- QUIK [15] employs a multi-stage k-mer filtering strategy combined with GPU acceleration, also specifically designed for barcode calling with extremely high throughput.

While prior work has compared subsets of these tools, existing benchmarks are limited in scope, focus primarily on simulated data, and do not systematically explore parameter sensitivity, hardware tradeoffs, or real empirical datasets. Here, we present a comprehensive benchmarking study of barcode decoding tools under conditions relevant to high-density spatial transcriptomics. Using both controlled simulations and real sequencing data from photolithographically synthesized arrays, we evaluate decoding accuracy, robustness to barcode length and library size, runtime performance, and scalability. Additionally, we provide a fully reproducible Nextflow-based pipeline [19] that integrates all evaluated barcode callers and supports both local execution and high-performance computing environments using a SLURM scheduler. Our goal is to provide practical guidance for selecting and configuring barcode decoding tools in the presence of high substitution and indel error rates.

## Methods

### Barcode generation and simulation of erroneous barcode reads

Barcode lengths of 28, 30, 32, 34, and 36 nucleotides were included in this benchmark because shorter barcodes provide limited sequence complexity under high substitution and indel error rates, making accurate decoding increasingly ambiguous as barcode libraries scale, whereas longer barcodes are expected to offer improved discriminability and robustness in high-density spatial transcriptomics settings. Candidate barcodes were generated as random integer arrays, each representing a nucleotide sequence, and were filtered to ensure compliance with a maximum homopolymer run constraint of 3, balanced G content ( 25%) and mean photolithographic printing cycles per nucleotide of 2.1. The filtered pool was then randomly shuffled and split into two equal subsets, so one subset could be used as a decoy set for precision analysis. Three barcode set sizes were generated: 21,000, 42,000, and 85,000 unique barcodes. These numbers were selected to mimic the number of spots that can be synthesized using our in-house photolithographic DNA printer [20]. Simulated datasets with known ground truth were generated to enable controlled evaluation of decoding accuracy. The Press (RandomBarcodes) error model [13] was used for this purpose. It applies errors in a non-iterative, position-independent manner. Starting from a barcode of length M, substitutions are applied by drawing from a binomial distribution and replacing selected positions with random bases, with a correction factor to account for substitutions that would otherwise leave a nucleotide unchanged. Deletions are applied by removing bases at randomly selected positions, and insertions are added at randomly selected positions in the shortened sequence. The resulting sequence is then truncated or padded to length M. This model assumes barcode start and end positions are not independently known at decoding time and is well-suited for large-scale simulation. Using this model, datasets of 200,000 and 1 million reads were generated from all barcode sets under three error regimes (Table 1).

**Table 1:** Error-rate regimes used in simulated barcode sequencing showing the relative contribution of substitution, insertion and deletion errors

Error level	Substitution rate	Insertion rate	Deletion rate
Low (18%)	2%	3%	13%
Medium (21%)	3%	4%	14%
High (32%)	3%	15%	14%

Exact error rates in photolithographic synthesis are highly dependent on synthesis parameter choices, particularly spot density, layout and photodeprotection exposure energy. Here, we base the error rates on those reported by Lietard et al. [9] and direct communication with the authors. The medium error rate (~23%) is expected for arrays printed with a checkerboard-like design where 1 in 2 spots are printed. According to the authors, reducing by half the number of spots (only 1 out of 4 spots printed) may reduce the error rate to ~20%. The authors envisage that printing the whole array (all spots printed) can significantly increase the insertion rate by a factor of ~3, as most insertions originate from diffracted and locally scattered light leading to leakage between adjacent spots. Unless otherwise stated, the low-error and 200,000-read datasets were used for analyses.

### Parameter calibration

To ensure fair and interpretable comparisons, each tool was evaluated near its optimal operating regime using systematic parameter calibration on simulated data with known ground truth. Only parameters directly controlling the precision–recall tradeoff were swept; all others were fixed based on preliminary analyses to avoid unnecessary over-tuning.

- Columba: identity threshold *I* (maximum edit distance)
- QUIK: k-mer length and rejection threshold *r* (maximum sequence-Levenshtein distance)
- RandomBarcodes: triage size $N_{\text {triage}}$ and rejection threshold $N_{\text {thresh}}$

Initial sweeps used 28-nt and 36-nt barcodes; intermediate lengths (30, 32, 34 nt) employed minimal calibration fixing secondary parameters (QUIK: 4-mer strategy; RandomBarcodes: Ntriage =100). Operating points prioritized high precision ($\ge 99.9\%$) while avoiding severe recall loss. Metrics: Precision = correctly assigned reads / total assigned reads; Recall = correctly assigned reads / total reads; Assignment rate = total assigned reads / total reads (includes incorrect assignments).

### Runtime benchmarking and scalability analysis

Runtime performance was evaluated after fixing tool-specific parameters at operating points selected to maintain high decoding accuracy (precision $\ge 99.9\%$). Benchmarks were conducted using the same simulated datasets as the initial calibration, consisting of 200,000 reads for barcode lengths of 28 nt and 36 nt. QUIK: Designed as a single-GPU algorithm, runs were performed with different GPU allocations to assess scheduling overhead and sensitivity to GPU provisioning. Although multiple GPUs could be allocated at the scheduler level, QUIK internally utilizes only one GPU. Two decoding strategies were evaluated: a single-stage 4-mer strategy and a two-stage 4+7-mer strategy. RandomBarcodes: All core decoding operations (k-mer triage and edit-distance verification) are executed on a single GPU. In addition, the workflow supports an external data-parallel execution model, in which input reads are split into independent subsets and processed concurrently, with each subset assigned to a separate GPU. Runtime benchmarks were therefore performed using 1, 2, and 4 GPUs by applying this external data-parallel strategy. Two representative triage configurations were evaluated: small triage window (Ntriage= 100) and large triage window (Ntriage= 5000). Although a similar approach would in principle be applicable to QUIK, we did not evaluate this strategy given QUIK’s low single-GPU runtime and the absence of such evaluation in the original QUIK study. Columba: Being CPU-based, strong scaling was evaluated by varying the number of CPU threads (1, 2, 4, 8, and 16 threads) while keeping all alignment parameters fixed. For all tools, wall-clock time, per-read processing time, and parallel speedup were recorded. Hardware: AMD EPYC 7413 24-Core Processor, NVIDIA A100-SXM4-80GB GPUs, 256 GB RAM. Software: Nextflow v25.04, Pytorch v2.1.2, CUDA v12.1.1, Cmake v3.26.3 Tool provenance: the QUIK version corresponding to the PNAS Nexus publication is tag v0.2.0, whereas the benchmarks in this study were performed using QUIK commit 74637f2c25 (full SHA 74637f2c258d16b3f3e00ce6e8e4e15e2b6e0e3c), obtained directly from the QUIK GitHub repository. Columba benchmarks used vanilla 2.0. RandomBarcodes currently has no formal tagged releases; therefore, all RandomBarcodes analyses were run from a fixed repository snapshot.

### Effect of barcode count on decoding performance

To assess robustness to library size, simulated datasets generated with 21,000, 42,000, and 85,000 barcodes for both 28-nt and 36-nt barcode lengths were used. Decoding was performed using fixed operating-point parameters, while only the rejection thresholds were varied to characterize precision–recall tradeoffs. Performance was summarized using assignment rate, precision, and recall. To characterise barcode-library separability, we additionally computed three distance metrics for each evaluated library: nearest-neighbour (NN) Hamming distance (distance from each barcode to its closest library member), sampled pairwise Hamming distance (from randomly sampled barcode pairs), and NN Levenshtein distance (computed on a subsample of 3,000 barcodes per library). Distances were analysed for all six benchmarked library configurations (21k, 42k, and 85k barcodes at 28 nt and 36 nt). Full distributions and summary statistics are provided in Additional file 3.

### Real sequencing data and decoy-based precision estimation

#### Printing of slides

A second-generation maskless digital photolithographic array synthesizer (MAS 2.0) was used to synthesize the DNA barcode libraries. This open-source device and synthetic approach, a modified form of standard phosphoramidite chemistry, have been previously described in detail [20–24]. Briefly, the MAS 2.0 consists of a 365 nm LED light source [25] that illuminates a UVA-optimized XGA digital micromirror device (DMD; Vialux V-7001 UV DLP Superspeed V-Module). The micromirrors of the DMD are tilted, under automated computer control, into one of two positions, ON or OFF. A diffraction-limited, unit magnification, catadioptric relay with a numerical aperture of 0.12 images the mirror pattern onto a photochemical synthesis flow cell. The pattern of UV light originating from the ON mirrors results in the selective removal of photolabile groups on DNA phosphoramidites, allowing the parallel synthesis of up to 786,432 oligonucleotides. The MAS 2.0 optical system is synchronized with an Expedite 8909 nucleic acid fluidics system that delivers phosphoramidites, reagents, and solvents to the flow cell. The DNA barcode libraries were synthesized 5’ to 3’ using 3’ BzNPPOC (benzoyl-2-(2-nitrophenyl)-propoxycarbonyl) DNA phosphoramidites (Orgentis Chemicals; #LV-4496, #LV-4495, #LV-4497, #LV-4473, #LV-4145). The photolabile BzNPPOC were removed using 45 s photoexposures with a radiant intensity of 120 mw/cm2, corresponding to a radiant exposure of 5.4 J/cm2, sufficient for *>95%* removal of the protecting groups [22, 25, 26]. The substrates used for the synthesis were aminosilane-coated glass slides (Schott Technical Glass Solutions, #1064875). After synthesis, the microarrays were immersed in a 1:1 (v/v) ethylenediamine/ethanol solution for 2 h at room temperature to remove the phosphodiester and nucleobase protecting groups, followed by washing twice with deionized water and drying with a stream of argon. Each glass slide was designed to contain four subarrays, each with features measuring 13.7 *× * 13.7 µm, with 1 µm spacing between adjacent features. Spatial barcode sequences were uniquely assigned to individual features, thereby encoding a unique spatial location on the slide. In this experiment, the four subarrays differed in probe density and design: one subarray was printed in quarter-density with 21,476 features, one subarray in half-density with 42,653 features, and two subarrays contained a full-density with 85,305 features. Of the two full-density subarrays, one contained probes with a cleavable site composed of a 20-base equimolar mixture of U- and T-phosphoramidites, while the second one contained a non-cleavable probe consisting of 20 T-phosphoramidites, serving as a negative control for cleavage. Probe sequences consisted of, from 5’ to 3’, a poly-T linker (5 nt), a cleavage site (20 nt), a Nextera Read 1 sequence (TCGTCGGCAGCGTCAGATGTGTATAAGAGACAG), a 10-nucleotide unique molecular identifier (UMI), a 36-nucleotide spatial barcode, and a Nextera Read 2 sequence (CTGTCTCTTATACACATCTCCGAGCCCACGAGAC). Following synthesis, slides were deprotected overnight at room temperature in a 1:1 (v/v) ethylenediamine:ethanol solution in a glass staining jar. Cleavage of probes from slide: Printed probes were released from the subarray by incubation with 50 *μ *L cleavage mixture, containing 5 U USER enzyme (New England Biolabs, #M5505) in 1X rCutSmart Buffer, per well of a multi-well hybridization cassette (Arrayit, #AHC1X16). The cleavage mixture was incubated overnight at $37^\circ$C with intermittent shaking (20 s of shaking, followed by 20 s of stationary). Following overnight incubation, the cleaved probes were collected into DNA LoBind tubes (Eppendorf, #EPP0030122208). To maximize probe recovery, the subarray was washed with 90 *μ *L nuclease free water, and the resulting wash solution was pooled with the primary recovery.

Library preparation: Probe purification was performed using Vivacon 500 centrifugal concentrators with a 10,000 MWCO membrane (Sartorius, #VN01H02). The columns were pre-washed with 200 *μ *L of nuclease-free water (pxlence, #PXL-PGW-1000) by centrifugation at 2500 x g for 10 min. Subsequently, the column is inverted to dry and centrifuged at 2500 x g for 2 min. The samples were diluted to a final volume of 500 *μ *L, loaded onto the columns, and centrifuged at 7400 x g for 25 min. Purified probes were eluted by transferring the columns to fresh tubes and centrifuging at 2500 x g for 3 min. All samples were finally adjusted to a uniform volume of 24 *μ *L.

For determination of the optimal number of indexing PCR cycles, 1 µL of each subarray sample was used as input in a 5 µL quantitative PCR (qPCR) reaction containing 1x PrimeTime Master Mix (IDT, #1055772), EvaGreen dye diluted to a final concentration of 1:40 (VWR, #31000-T ), and 250 nM of each Nextera-compatible indexing primer (Table1). qPCR was carried out on a LightCycler 480 instrument (Roche) with an initial enzyme activation step of 3 min at 95 $^\circ$C, followed by 45 cycles of 15 s at 95 $^\circ$C and 1 min at 60 $^\circ$C, and a final extension step of 2 min at 72 $^\circ$C. The optimal number of indexing PCR cycles was defined as the quantification cycle (Cq) corresponding to 50% of the maximum fluorescence signal, minus four cycles.

The remaining 23 *μ *L of each pre-amplified library was subsequently indexed using 1x PrimeTime Master Mix and 250 nM of each indexing primer (Table 2) in a total reaction volume of 57.5 *μ *L, using the previously determined optimal number of PCR cycles. Indexed libraries were purified using a 1.8x Ampure XP bead cleanup (Beckman Coulter, #A63881) and eluted in a final volume of 15 *μ *L. Libraries were pooled equimolarly to the lowest-concentration sample, as determined by KAPA Library Quantification qPCR (Roche, #KK4854) using the average fragment length between 50 and 1450 bp assessed by Fragment Analyzer (Agilent).

**Table 2:** Sequences of unique P5- and P7-indexes used for PCR amplification of cleaved probes

Index	Sequence
A1-UDI-001-P7	CAAGCAGAAGACGGCATACGAGATACTCTCGAGTCTCGTGGGCTCGGAGATGTGTATAAGAGACAG
B1-UDI-013-P7	CAAGCAGAAGACGGCATACGAGATATCCAGAGGTCTCGTGGGCTCGGAGATGTGTATAAGAGACAG
C1-UDI-025-P7	CAAGCAGAAGACGGCATACGAGATCGTTGCAAGTCTCGTGGGCTCGGAGATGTGTATAAGAGACAG
D1-UDI-037-P7	CAAGCAGAAGACGGCATACGAGATATCGCCATGTCTCGTGGGCTCGGAGATGTGTATAAGAGACAG
A1-UDI-001-P5	AATGATACGGCGACCACCGAGATCTACACTGGTACAGTCGTCGGCAGCGTCAGATGTGTATAAGAGACAG
B1-UDI-013-P5	AATGATACGGCGACCACCGAGATCTACACCGACGTTATCGTCGGCAGCGTCAGATGTGTATAAGAGACAG
C1-UDI-025-P5	AATGATACGGCGACCACCGAGATCTACACCGCTCTATTCGTCGGCAGCGTCAGATGTGTATAAGAGACAG
D1-UDI-037-P5	AATGATACGGCGACCACCGAGATCTACACGTTACGCATCGTCGGCAGCGTCAGATGTGTATAAGAGACAG

#### Sequencing

The final libraries were quality-checked using a Fragment Analyzer and quantified using the KAPA Library Quantification qPCR kit prior to sequencing. Sequencing was performed on a MiSeq Micro flow cell (Illumina, #MS-103-1002) using 46-8-8-46 cycle chemistry, at a loading concentration of 6 pM and a 5% PhiX control spike-in. Sequencing yielded 420,338 reads for the full-density subarray, 526,931 reads for the half-density subarray, and 657,529 reads for the quarter-density subarray. Raw FASTQ files were directly used for barcode calling, performed with operating-point parameters selected from simulation-based calibration:

- QUIK: 4-mer, *r = 8*
- RandomBarcodes: $$N_{\text {thresh}} = 9$$, $$N_{\text {triage}} = 100$$
- Columba: 21k with *I = 77*; 42k and 85k with *I = 80*

Because true barcode assignments are unknown for empirical data, recall cannot be computed and assignment rate alone is insufficient to assess call quality. Precision was therefore estimated using a normalised decoy-based strategy in which additional barcodes not present on the subarray were appended to the reference set and assignments to decoy barcodes were counted as false positives. Based on a sensitivity analysis (Additional file 2), decoys were included at 50% of the real reference-set size (decoy:real = 1:2), which provided stable precision estimates with minimal perturbation of decoding behaviour.

#### Error rate effect

The three tools were evaluated under three error rates described above. Tests were conducted across barcode libraries of 21,000, 42,000, and 85,000 unique 36-nt barcodes with 200,000 simulated reads. For RandomBarcodes, we set $N_{\text {thresh}} = 9$ and $N_{\text {triage}} = 100$. For QUIK, we used the 4-mer strategy and rejection threshold *r = 8*. For Columba, the identity threshold was set to *I = 80*.

#### Benchmarking pipeline and reproducibility

To support reproducibility and portability, all benchmarking experiments were implemented using a custom Nextflow pipeline (https://github.com/OncoRNALab/BarcodeCalling_benchmark). The pipeline provides a unified interface for running QUIK, RandomBarcodes, and Columba and can be executed both locally and on high-performance computing systems using a SLURM executor. The pipeline allows users to select the barcode calling tool and specify tool-specific parameters through configuration files, ensuring consistent execution across experiments. As input, the pipeline accepts paired FASTQ files and a plain text file containing one barcode per line. This framework enables systematic parameter sweeps, standardized metric computation, and fully reproducible benchmarking across diverse hardware environments.

## Results

### Parameter calibration

**Fig. 1:**
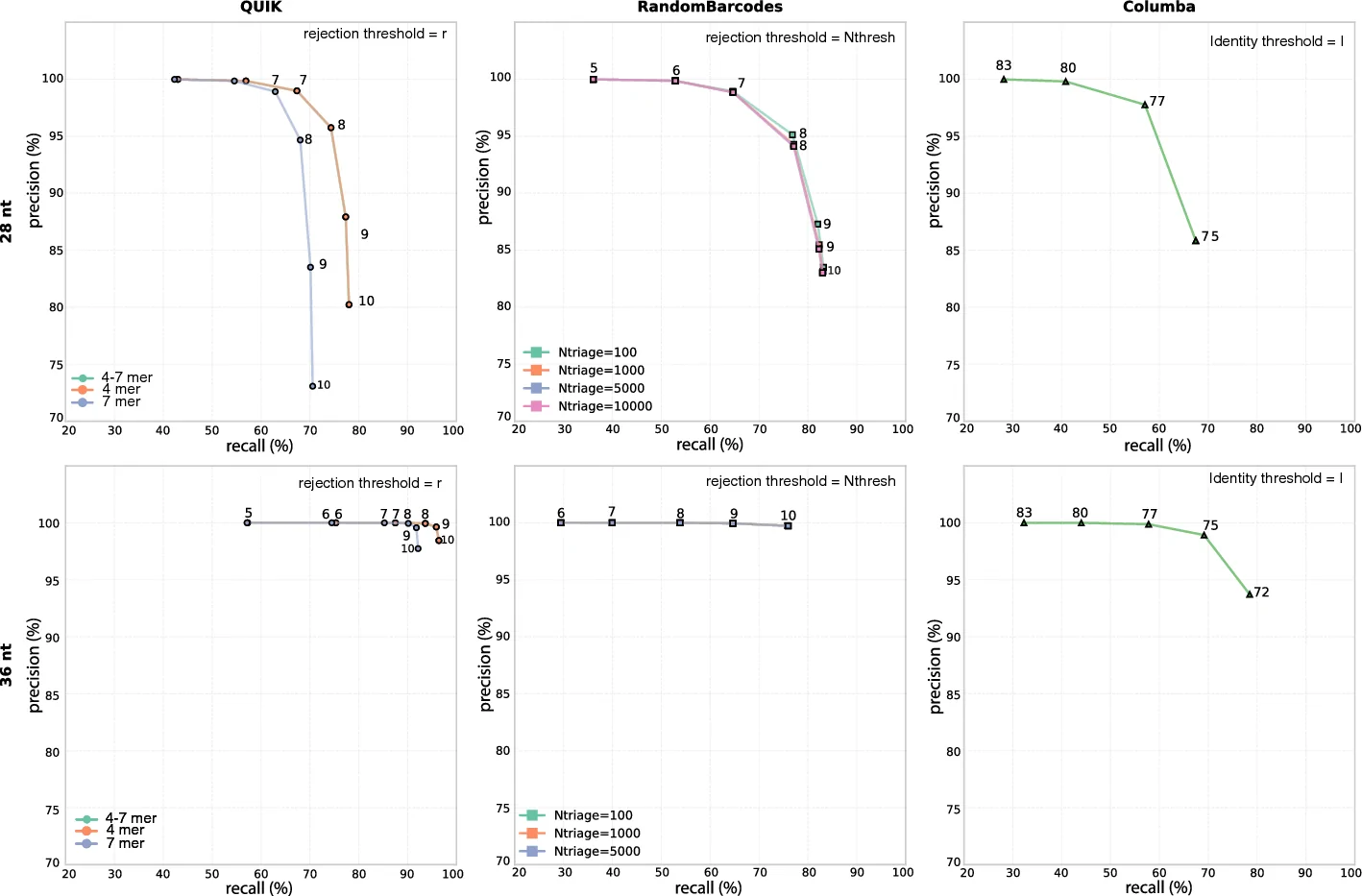
Parameter calibration for 28 nt and 36 nt barcodes. Precision–recall curves obtained by sweeping the primary rejection (or identity) threshold for each of the three barcode callers. For QUIK, three k-mer strategies were evaluated, while for RandomBarcodes four triage sizes were tested; for Columba, only the identity threshold was varied. In all cases, the rejection or identity threshold determines the trade-off between precision and recall. For RandomBarcodes, the triage size (Ntriage) had minimal impact on accuracy metrics, whereas QUIK achieved higher precision and recall using the 4-mer and 4+7-mer strategies compared with the 7-mer strategy alone. For Columba, increasing recall beyond ~60% resulted in a pronounced loss of precision

For 28-nt barcodes, all three tools exhibited a clear tradeoff between precision and recall as rejection thresholds were tightened (Fig. 1).

- For Columba, a precision of 99.8% and recall of 40.8% was obtained at *I = 80*. A precision of 99.99% was only achievable at *I = 83*, but with substantial loss of recall (~28%).
- For QUIK, at *r = 5*, all strategies achieved precision above 99.9% (crucial for spatial transcriptomics applications) and recall ~42%, with the 4-mer strategy yielding slightly higher recall. However, if lower precision is acceptable (~95%), recall of ~74% can be achieved at *r = 8*.
- For RandomBarcodes, the number of barcodes surviving triage ($N_{\text {triage}}$) had little effect on metrics. Precision above 99.9% was obtained with all three $N_{\text {triage}}$ values combined with $N_{\text {thresh}} = 5$, with recall of 36.1%. Recall increased to 64.7% if a precision of 98.9% is accepted by setting $N_{\text {thresh}} = 7$.

For 36-nt barcodes, all tools demonstrated improved discriminative power, achieving higher precision at less aggressive rejection settings (Fig. 1).

- For Columba, the best precision (99.99%) was obtained at *I = 80*, with recall of 44.02%. An increase of ~13% in recall can be achieved by setting *I = 77* while maintaining precision around 99.87%.
- For RandomBarcodes, as observed with 28-nt barcodes, $N_{\text {triage}}$ had practically no effect on accuracy metrics. A moderate triage size ($N_{\text {triage}} = 100$) combined with $N_{\text {thresh}} = 8$ achieved precision of 99.99% with recall 53.8%. An increase of 10% in recall was achieved with $N_{\text {thresh}} = 9$, while maintaining high precision of 99.95%.
- For QUIK, perfect precision was obtained with recall of 57.3% for all three strategies at *r = 5*. Furthermore, setting *r = 7* dramatically increased recall to 87.5% while maintaining precision above 99.99%.

**Fig. 2:**
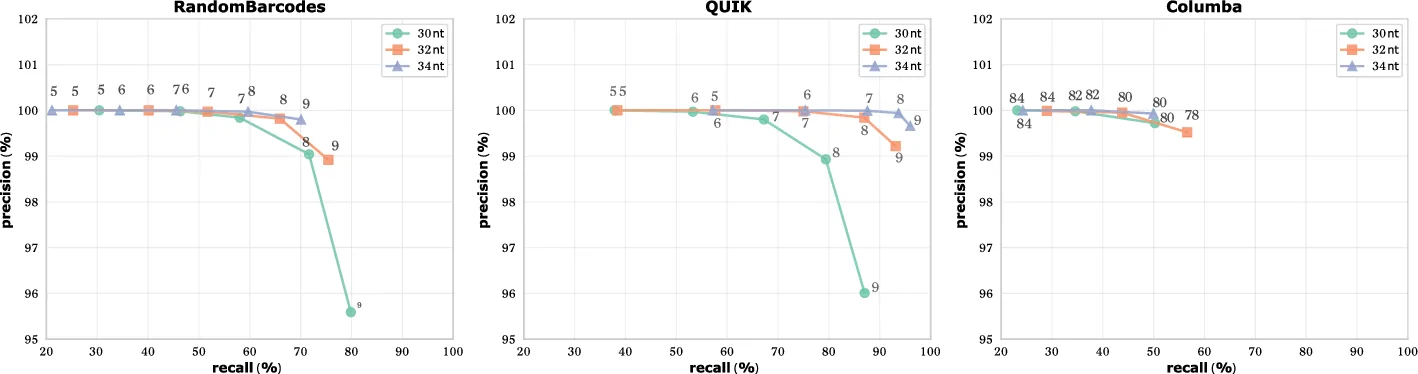
Parameter calibration for intermediate barcode lengths (30–34 nt). Precision–recall curves for barcodes of length 30, 32, and 34 nt obtained by varying the rejection threshold for each tool. The 4-mer strategy and Ntriage = 100 were fixed for QUIK and RandomBarcodes, respectively. Increasing barcode length consistently improved the precision–recall trade-off, enabling higher and more stable precision across all three callers

For intermediate lengths (30–34 nt), QUIK with the 4-mer strategy and *r = 8* achieved 99.97%/53% at 30 nt, 99.99%/58% at 32 nt, and 99.97%/75% at 34 nt. RandomBarcodes ($N_{\text {triage}} = 100$) achieved 99.95%/46% at 30 nt, 99.95%/52% at 32 nt, and 99.95%/60% at 34 nt. Columba maintained $\ge 99.93\%$ precision at all three lengths, while recall increased with length from 34.58% (30 nt) to 43.81% (32 nt) and 49.90% (34 nt) (Fig. 2).

### Runtime performance and scalability

**Fig. 3:**
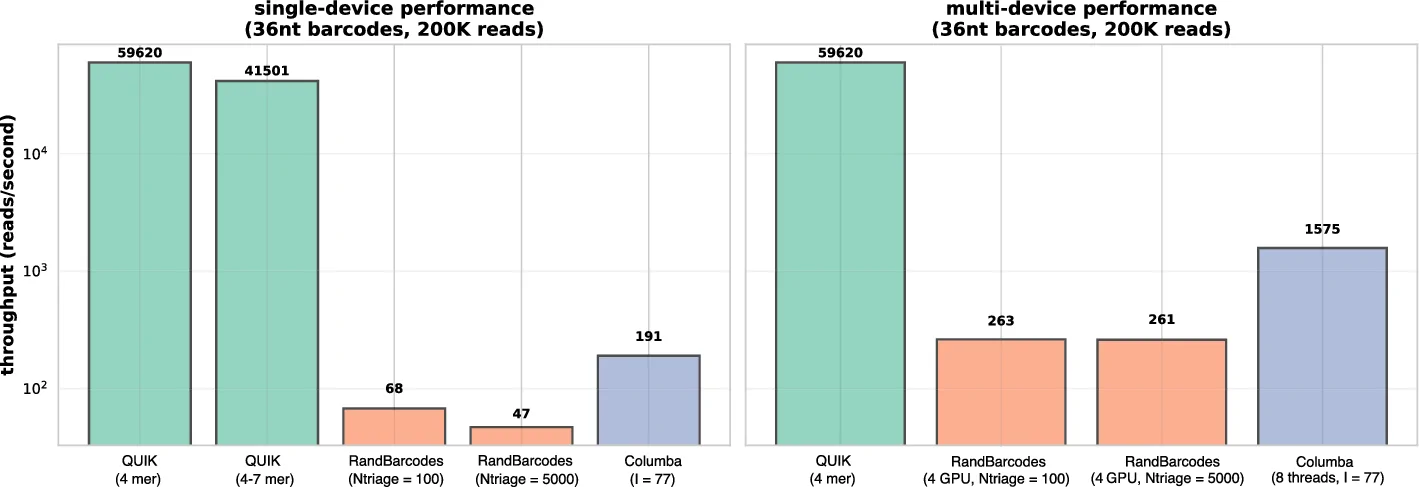
Runtime performance. Bar plots showing throughput in reads per second for each barcode caller using **A** a single compute device and **B** multiple devices. QUIK, using a single GPU and the 4-mer strategy, achieved substantially higher throughput than both multi-GPU RandomBarcodes (4 GPUs) and multi-CPU Columba (8 threads)

**Fig. 4:**
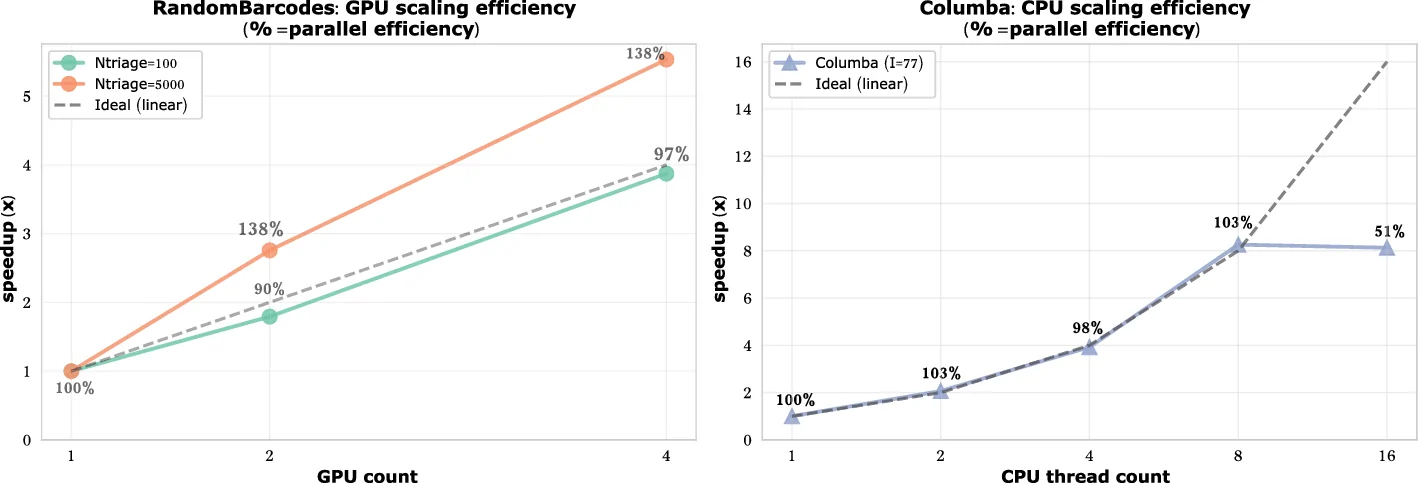
Scaling efficiency. Speedup curves for RandomBarcodes and Columba as computational resources were increased. RandomBarcodes was evaluated using 1, 2, and 4 GPUs with two triage sizes (Ntriage = 100 and 5000), while Columba was benchmarked with a fixed identity threshold (-I = 77) and 1, 2, 4, 8, and 16 CPU threads. Both tools exhibited near-linear scaling. RandomBarcodes with Ntriage = 5000 showed the largest gains, with an average speedup of approximately 138% per doubling of GPU count. Columba scaled linearly up to 8 threads, after which additional threads provided diminishing returns, likely due to insufficient workload to saturate further parallelism

All runs used the 21,000-barcode set of 36-nt barcodes and 200,000 simulated reads (Figs. 3 and 4).

- Columba: Processed 190 reads per second using 1 thread, showing near-linear CPU scaling up to 8 threads where it reached 1,550 reads per second.
- RandomBarcodes: Triage size clearly affected runtime: 67.9 reads per second processed with $N_{\text {triage}} = 100$ and 47.2 reads per second with $N_{\text {triage}} = 5000$. RandomBarcodes exhibited effective multi-GPU scaling with speedup using 4 GPUs of 3.87*× * using $N_{\text {triage}} = 100$ and 5.53*× * with $N_{\text {triage}} = 5000$.
- QUIK: The 4-mer strategy was ~30% faster than 4+7-mer, with 59,620 and 41,500 decoded reads per second, respectively. While RandomBarcodes took ~30 min and Columba ~18 min to decode 200,000 reads, QUIK outperformed both with total wall-clock time of 3.35 s.

The observed runtime advantage of the 4-mer strategy over 4+7-mer differs from the original QUIK publication and is consistent with software-version differences between studies. Specifically, the published QUIK results correspond to tag v0.2.0, while our analyses used a newer GitHub development snapshot. In additional large-library benchmarks (Additional file 1), the 4-mer strategy remained consistently faster than 4+7-mer across all tested barcode-set sizes, with no meaningful gain in precision or recall from the two-stage strategy under the evaluated conditions.

**Fig. 5:**
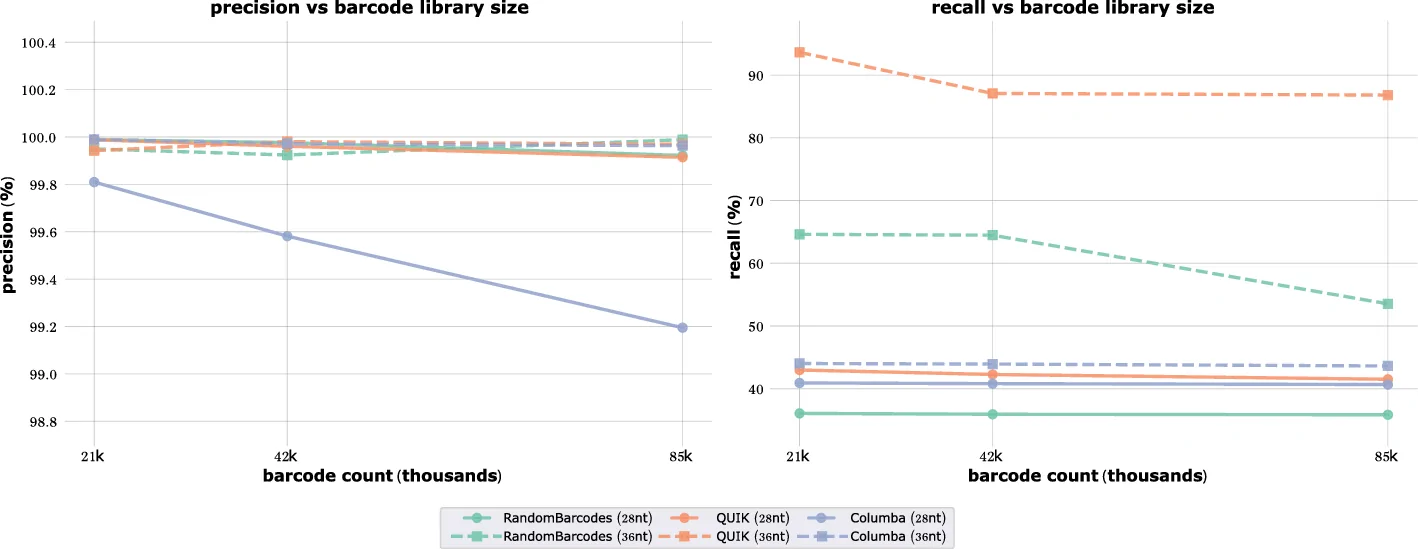
Effect of barcode library size. Line plots showing (A) precision and (B) recall for the three callers across barcode libraries of 21k, 42k, and 85k barcodes at lengths of 28 nt and 36 nt. Precision remained high and near 100% for all tools except Columba with 28 nt barcodes, where precision decreased as barcode library size increased. The highest recall was consistently achieved by QUIK using 36 nt barcodes

Overall, QUIK achieved the best runtime by a wide margin, followed by multi-threaded Columba and RandomBarcodes in the multi-GPU setting, whereas single-GPU RandomBarcodes remained substantially slower than all other configurations. Effect of barcode length and library size Using a stringent operating-point criterion (precision $\ge 99.5\%$ where possible, otherwise the highest-precision configuration), increasing barcode count (21k *→ * 42k *→ * 85k) had only a modest impact on performance compared with barcode length and algorithm. RandomBarcodes maintained near-perfect precision across all library sizes (99.92–99.99%), but recall remained low for 28-nt barcodes (~35.9–36.1%) and increased substantially for 36-nt barcodes (~64.5% at 21–42k), before dropping at 85k (53.5%) (Fig. 5), indicating reduced sensitivity at the largest barcode library. QUIK achieved the highest recall while preserving $\ge 99.9\%$ precision for all conditions: recall was ~42% for 28-nt barcodes and rose sharply for 36-nt barcodes (93.6% at 21k; 86.8–87.1% at 42–85k), with optimal thresholds becoming slightly more conservative as barcode count increased (*r = 8* at 21k versus *r = 7* at 42–85k) (Fig. 5). Columba showed intermediate recall (~40.7–40.9% for 28 nt; ~43.6–44.0% for 36 nt) with precision between 99.2–99.8% for 28 nt and 99.96–99.99% for 36 nt, and comparatively little change in recall with barcode count (Fig. 5). Overall, these results indicate that increasing barcode library size from 21k to 85k does not substantially affect recall for the k-mer–based methods at fixed high precision, whereas barcode length is the dominant factor, and QUIK consistently provides the best recall at stringent precision.

Although the primary benchmark focused on libraries up to 85k barcodes, we additionally evaluated larger libraries (170k, 340k, and 786k) using simulated 36-nt datasets at medium error rate (1 M and 4 M reads; full results in Additional file 1). QUIK remained the most sensitive caller but showed decreasing precision and recall as library size increased (e.g., precision 99.2% to 97.1% and recall 86.0% to 82.5% at 1 M reads), indicating that at high library densities the rejection threshold should be made more stringent; for the 786k setting, values below *r=8* are likely preferable to recover precision. Columba maintained high precision but exhibited non-linear runtime scaling, with a marked increase in per-read time at 786k barcodes.

**Fig. 6:**
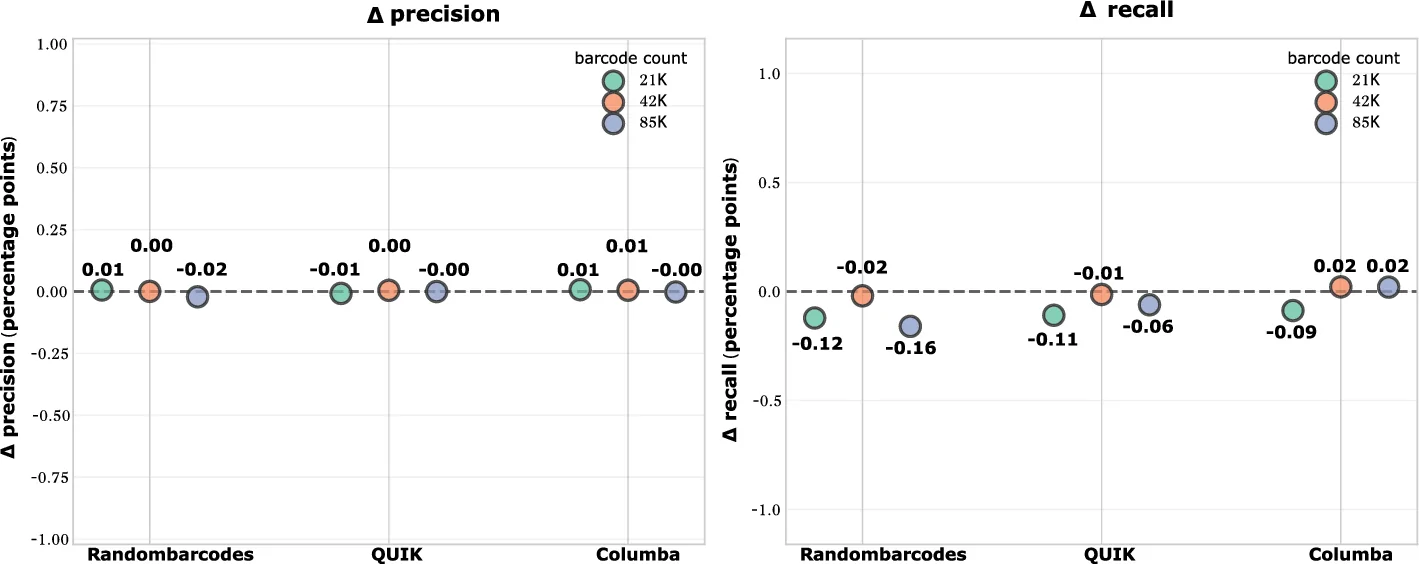
Scaling with dataset size. Differences in (A) precision and (B) recall between decoding 200,000 and 1,000,000 reads. Across all tools and barcode library sizes, both precision and recall remained highly stable, indicating that decoding accuracy is largely insensitive to dataset size within this range

Similarly, scaling from 200,000 to 1 million reads (using the medium-error simulation) produced minimal changes in accuracy across all tools, indicating stable operating behavior at higher depth. RandomBarcodes maintained near-perfect precision (~99.8–99.95%) with recall ~56% and only negligible recall shifts. QUIK likewise remained stable, preserving high precision (~99.6–99.9%) and high recall (~87–89%), with only small recall decreases at 1 M. Columba is also stable: precision stays uniformly high at both depths (~99.94–99.98%) and recall remains ~35.4–35.5% with only marginal changes (Fig. 6). Overall, scaling from 200,000 to 1 million reads did not change the relative ranking of methods and performance metrics were largely stable.

### Distance properties of benchmark barcode libraries

To assess intrinsic sequence separability of the benchmark libraries, we analysed NN Hamming distance, sampled pairwise Hamming distance, and NN Levenshtein distance across all six library configurations (21k, 42k, and 85k at 28 nt and 36 nt). For 28-nt libraries, median NN Hamming distance was 11 (21k), 10 (42k), and 10 (85k), with minima of 6, 6, and 5, respectively. For 36-nt libraries, median NN Hamming distance was 16 (21k), 15 (42k), and 15 (85k), with minima of 10, 10, and 9. Pairwise Hamming distributions were broader and shifted to higher values (median 21 for 28 nt and 27 for 36 nt), indicating that randomly selected barcode pairs are substantially more divergent than nearest neighbours. NN Levenshtein distances followed the same trend but were typically one to two positions lower (median 10 for 28 nt and 14 for 36 nt), consistent with the additional flexibility of insertion/deletion operations. Although minimum NN distances decreased slightly with larger library size, all libraries retained substantial separation under the evaluated conditions. Detailed distributions are shown in Additional file 3.

### Error rate effect

**Fig. 7:**
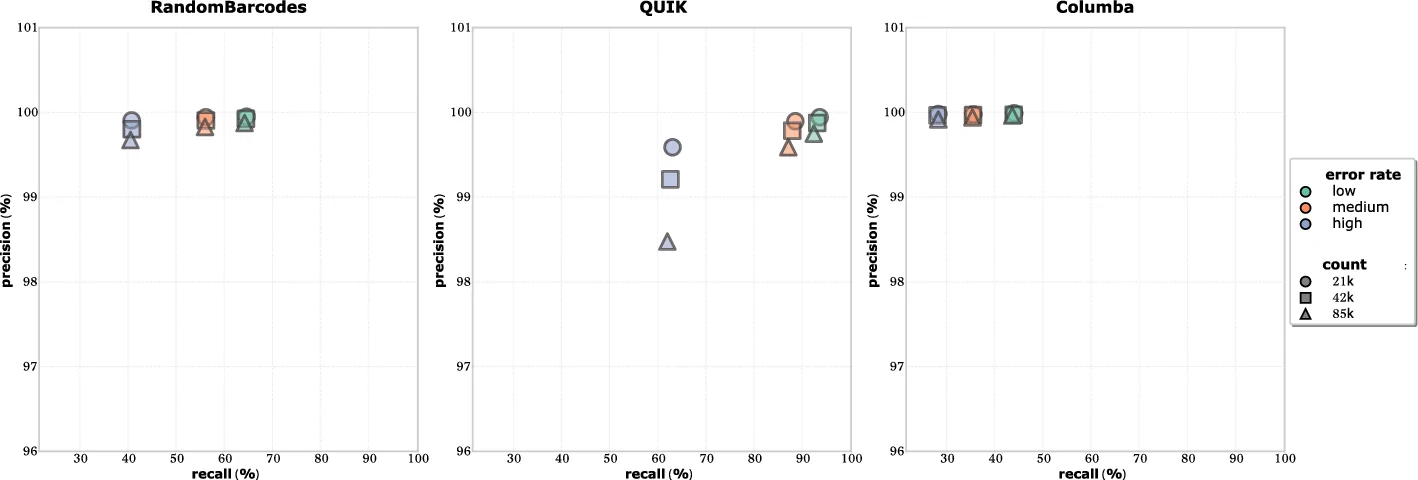
Effect of error rate. Precision–recall curves for the three callers and three barcode library sizes under low (18%), medium (21%), and high (32%) error regimes. Precision for RandomBarcodes and Columba was largely robust across error rates. In contrast, QUIK showed a reduction in precision at high error rates for the largest barcode library (85k). Despite this, QUIK consistently achieved higher recall than the other tools, indicating that precision could be recovered by applying more stringent rejection thresholds

To assess robustness to sequencing/synthesis noise, we benchmarked RandomBarcodes, QUIK, and Columba on simulated datasets spanning low, medium, and high error regimes across barcode libraries of 21,000, 42,000, and 85,000 unique 36-nt barcodes. RandomBarcodes maintained near-perfect precision across all conditions (99.67–99.95%) but showed a strong error-dependent loss of recall, dropping from ~64.2–64.6% (low) to ~56.0–56.2% (medium) and ~40.5–40.8% (high), with little dependence on barcode count (Fig. 7). QUIK combined similarly high precision (98.48–99.94%) with substantially higher recall than the other tools; recall remained ~92.4–93.6% under low error, declined modestly to ~87.1–88.6% under medium error, and dropped to ~62.0–63.1% under high error, with only a small decrease at larger barcode sets (Fig. 7). Columba maintained uniformly high precision across barcode counts and error regimes ($\ge 99.96\%$ in all cases), while recall decreased monotonically with increasing error rate—from ~43.6–44.0% (low) to ~35.5% (medium) and ~28.1–28.3% (high) (Fig. 7). Overall, increasing error rates primarily reduced recall for all tools; QUIK consistently retained the highest recovery at high precision, RandomBarcodes prioritized maximal precision at the expense of recall, and Columba remained highly precise but substantially less sensitive, with recall further declining as errors increased.

### Performance on real sequencing data

**Fig. 8:**
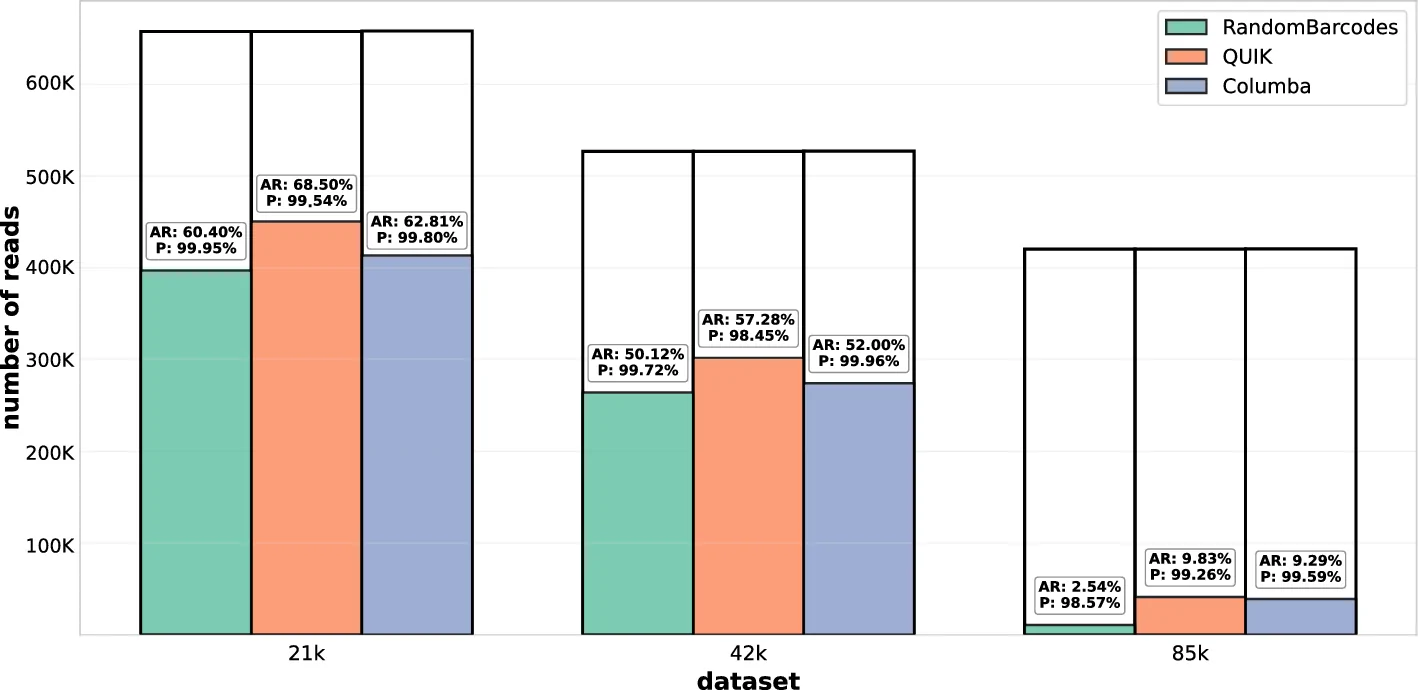
Performance on real empirical data. Bar plots showing the total number of sequenced reads and successfully decoded reads for each tool on real data generated from photolithographically synthesised arrays with varying spot densities. Precision (P) was estimated using the normalised decoy-based approach with a 50% decoy-to-real reference ratio (reported above each bar). Assignment rate (AR) decreased strongly with array density for all tools, with QUIK showing the highest AR at 21k and 42k and comparable AR to Columba at 85k

Across photolithographically synthesised in situ barcode subarrays, normalised decoy-based precision was high for all three tools at every density (98.5–100%), but assignment rate decreased strongly with increasing array density (21k *→ * 42k *→ * 85k; Fig. 8). QUIK consistently achieved the highest assignment rate (68.5%, 57.3%, and 9.8% at 21k, 42k, and 85k, respectively), followed by Columba (62.8%, 52.0%, and 9.3%) and RandomBarcodes (60.4%, 50.1%, and 2.5%). Precision remained *≥ *99.3% for QUIK and Columba at 21k and 85k; at 42k, QUIK precision was 98.5% while Columba reached 100.0% (Additional file 2).

**Fig. 9:**
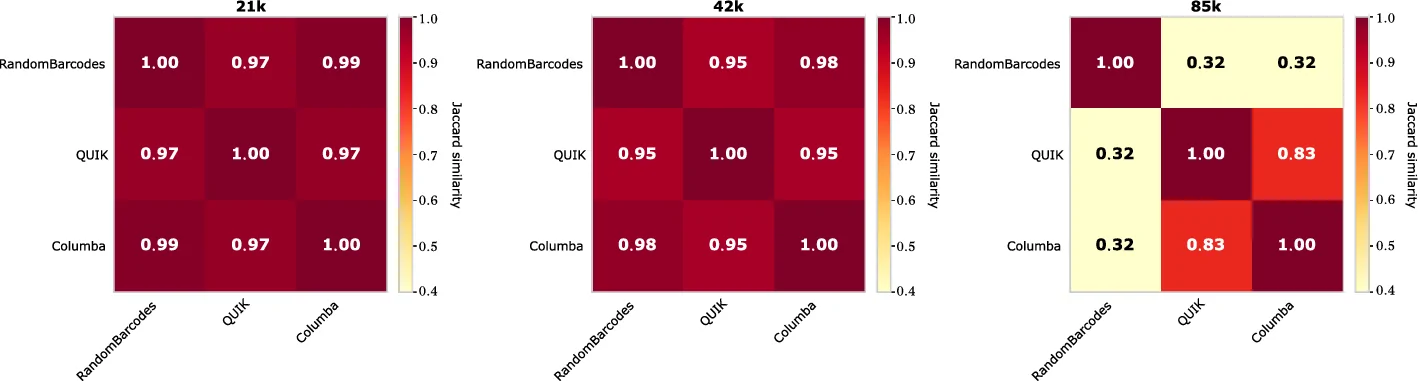
Barcode overlap across tools. Heatmaps showing pairwise Jaccard similarity of barcode sets recovered by each caller for the three real datasets. At 85k density, overlap between tools was reduced despite substantial shared recovery between QUIK and Columba (23,850 barcodes), with additional tool-specific recovery (2,899 unique to QUIK and 1,834 unique to Columba), supporting partial complementarity at high density

At the 85k full-density subarray, barcode recovery diverged substantially between tools (Fig. 9). QUIK recovered the greatest number of unique barcodes (26,861; 31.5% of the 85,305-barcode reference), followed by Columba (25,697; 30.1%) and RandomBarcodes (8,502; 10.0%). Overlap between QUIK and Columba was substantial (23,850 shared barcodes), yet each recovered barcodes missed by the other (2,899 unique to QUIK and 1,834 unique to Columba). The union of QUIK and Columba covered 28,583 unique barcodes (33.5% of the reference), compared with 31.5% for QUIK alone and 30.1% for Columba alone.

## Discussion

This comprehensive benchmarking study evaluated three distinct computational approaches to DNA barcode decoding under error conditions representative of photolithographic synthesis and high-density spatial transcriptomics. Overall, the methods exhibit clear trade-offs between recovery, precision, and computational cost, which should be matched to the intended application. QUIK consistently provided the best overall balance, achieving the highest recall while maintaining high precision (*>99.5%*) and running at very high throughput (~59,620 reads/s on a single GPU). RandomBarcodes was the most conservative caller, reaching near-perfect precision (*>99.9%*) at the cost of substantially lower recall (~56%) and markedly slower throughput at the evaluated operating point (~68 reads/s). Columba, evaluated here as a lossless approximate matching approach, remained a practical CPU-only alternative with respectable throughput (~1,550 reads/s at 8 threads), achieving very high precision on the simulated 36-nt benchmarks (typically $\ge 99.9\%$), albeit with substantially lower recall than QUIK. Because barcode-calling software is under active development, cross-study runtime comparisons should be interpreted in the context of the exact tool version used, as implementation updates can change strategy-level performance rankings. QUIK’s k‑mer filtering strategy operates under a different philosophy than classical error-correcting codes or exhaustive alignment. By precomputing k‑mer occurrence lists and using positional information to generate a compact candidate set, QUIK leverages redundancy in local sequence features that persists despite substitutions and moderate indel rates. This design explains both its high recall at stringent precision thresholds and its large runtime advantage over approaches that require heavier per-read verification. The strong improvement in performance with increasing barcode length (28 *→ * 36 nt) is consistent with increased sequence complexity and reduced near-collisions in large libraries. For the k‑mer–based methods, longer barcodes provide more informative k‑mer anchors and improve candidate discrimination, translating into substantially higher recall at similar precision thresholds. Real-data analyses further highlight that the main limitation at higher array density is loss of sensitivity/assignment rather than loss of precision. Across the 21k, 42k, and 85k photolithographic arrays, decoy-based estimates indicate that precision remains high (~99.8–100%) for all tools, while assignment decreases sharply with density. At the 85k full-density subarray in particular, barcode recovery becomes strongly method-dependent: QUIK recovered 26,861 barcodes in total, followed by Columba (25,697 total) and RandomBarcodes (8,502 total), while 2,899 barcodes were unique to QUIK and 1,834 were unique to Columba. The normalised decoy-based precision for Columba at 85k (99.6%) supports that many of these additional calls are not driven by widespread spurious matching, and instead reflect complementary recovery of difficult barcodes missed by k-mer filtering. Consistent with this complementarity, the union of QUIK and Columba covers 28,583 barcodes (33.5% of the 85,305 reference set), motivating ensemble strategies when maximizing barcode coverage is a priority. This complementarity reflects fundamental algorithmic differences. k-mer methods require sufficient k-mer anchors to survive errors for initial candidate filtering. When synthesis errors cluster adjacently in the same barcode all overlapping k-mers in that sequence may be destroyed, causing complete triage failure even when the overall edit distance remains decodable. Columba’s exhaustive search schemes explore all alignment paths within edit distance *k*, enabling recovery through alternative routes in the dynamic programming matrix that bypass corrupted regions.

### Practical recommendations

Based on both simulated and empirical data, QUIK is the preferred primary caller across all evaluated array densities. On real datasets using the normalised decoy-based approach, QUIK achieved assignment rates of 68.5%, 57.3%, and 9.8% for the 21k, 42k, and 85k arrays, respectively. Normalised decoy-based precision was 99.5% (21k), 98.5% (42k), and 99.3% (85k) for QUIK, and 99.8%, 100.0%, and 99.6% for Columba at the same densities. For 36-nt barcodes, practical operating points are obtained with the 4-mer strategy and rejection thresholds in the range *r = 7*–8.

When maximal barcode recovery is required, an ensemble of QUIK and Columba should be considered, particularly for high-density arrays (*≥ 85*k spots). At 85k, Columba recovered 1,834 unique barcodes not detected by QUIK, and normalised decoy-based precision (99.6%) supports that these additional calls are reliable. The union of QUIK and Columba covered 28,583 unique barcodes (33.5% of the 85,305-barcode reference), compared with 26,861 (31.5%) for QUIK alone. This gain must be balanced against runtime: at 85k density, per-read runtimes were approximately 0.09 ms for QUIK and 0.33 ms for Columba.

The sharp drop in assignment rate at 85k density (from ~57% at 42k to ~10% at 85k) indicates that synthesis quality, rather than barcode design or decoder choice, becomes the dominant bottleneck at full array density. Our results support the use of 34–36 nt barcodes when total error rates exceed 20%, although the substantial assignment drop observed at 85k despite 36-nt barcodes indicates that synthesis conditions can become the dominant limitation. In this regime, optimising synthesis strategy (for example, checkerboard-like or quarter-density layouts) may yield larger practical gains than further barcode length increases. Finally, we recommend using the combined decoy approach with 25–50% decoys as a routine quality check to obtain a self-consistent precision estimate without requiring ground truth; operating points calibrated on simulations should still be validated on pilot real datasets before large-scale use.

### Limitations of the current study

This study has several limitations that should be considered when interpreting the results. First, although the simulations captured global error-rate regimes, they did not model systematic spatial artifacts or local error correlations between adjacent spots. This simplification may contribute to the performance gap observed between simulations and the real 85k arrays.

Second, the empirical sequencing depth was modest (~1.6 million reads across all three libraries). While this depth is sufficient to estimate assignment rate and decoy-based precision, it may be insufficient for robust estimation of barcode coverage, defined as the fraction of unique reference barcodes recovered.

Third, runtime benchmarking was performed on a specific hardware and software stack (GPU/CPU architecture, CUDA, and PyTorch versions). Absolute runtimes and relative speedups may therefore shift on other systems, even if the overall performance ranking remains similar.

Finally, the benchmark focused on randomly generated barcode libraries constrained by homopolymer limits, balanced G content, and approximately 2.1 printing cycles per added nucleotide. Barcode sets designed with explicit minimum-distance constraints (for example, ECC-based designs) may exhibit different decoding behavior, particularly for alignment-based callers such as Columba. In addition, although QUIK can operate in CPU-only mode according to the original publication, we restricted our evaluation to the GPU implementation.

## Conclusions

This benchmarking study demonstrates that k-mer filtering approaches, particularly QUIK, have fundamentally shifted the performance frontier for DNA barcode decoding under high error rates. The ability to accurately decode 87% of reads at 99.99% precision with 36-nt random barcodes at 20% error rates would have been considered impossible with traditional ECCs, which typically support only 1–2 error corrections in libraries of $<10^4$ barcodes. The success of QUIK validates the theoretical prediction in Theorem 1 of Uphoff et al. [15] that k-mer-based filtering can reduce computational complexity by orders of magnitude without sacrificing accuracy, effectively making "brute-force" comparison of millions of reads against millions of barcodes practical on commodity hardware. Critically, barcode coverage analysis revealed that Columba detects a substantially different barcode subset than k-mer methods, particularly at high array densities where it uniquely recovered 1,834 barcodes not recovered by QUIK . This orthogonality suggests that ensemble approaches combining QUIK (for speed and precision) with Columba (for complementary coverage) could improve barcode recovery in challenging high-density applications, though at increased computational cost. For the spatial transcriptomics community, these results provide a clear path forward: 34–36 nt random barcodes decoded with QUIK enable high-density arrays ($>85{,}000$ spots) while maintaining the precision necessary for confident spatial mapping. The open-source Nextflow pipeline provided with this study ensures these methods are accessible and reproducible across research groups. As photolithographic synthesis continues to improve and error rates decline, the performance advantage of longer barcodes and sophisticated decoding algorithms will only increase, enabling ever-larger spatial atlases of tissue biology.

## Supplementary Information

Additional file 1: Title: Extended scalability benchmarking of barcode callers on large simulated libraries. Description: Detailed results for simulated 36-nt libraries at medium error rate for170k, 340k, and 786k barcodes (1M and 4M reads), including assignment rate, precision, recall, and runtime for QUIK, RandomBarcodes, and Columba, and comparison of QUIK 4-mer versus 4+7-mer strategies under fixed thresholds

Additional file 2: Title: Normalised decoy-based precision: sensitivity analysis and updated real-data summary. Description: Sensitivity of precision estimates to decoy fraction (25–50% relative to the real reference set)

Additional file 3: Title: Distance properties of benchmark barcode libraries. Description: Nearest-neighbour Hamming, sampled pairwise Hamming, and nearestneighbourLevenshtein distance distributions for the six evaluated libraries (21k, 42k,and 85k barcodes at 28 nt and 36 nt), with figures and summary statistics referencedin the main text

## Data Availability

The raw FASTQ files and simulated data generated in this study have been deposited in the Zenodo repository and are available at the following URL: https://doi.org/10.5281/zenodo.18387161. Tools: QUIK (https://github.com/uni-halle/quik), RandomBarcodes (https://github.com/whpress/RandomBarcodes), Columba (https://github.com/biointec/columba). Source code, documentation, and guides to reproduce the results can be accessed at https://github.com/OncoRNALab/BarcodeCalling benchmark.
