# Supplementary material for: Benchmarking DNA barcode decoding strategies under high error rates
Source: BMC Bioinformatics. 2026 Jun 23;27:213. doi: 10.1186/s12859-026-06540-x (PMC13551773; doi:10.1186/s12859-026-06540-x)
Supplement: Supplementary file 1 [file 12859_2026_6540_MOESM1_ESM.pdf]

Additional File 1

1. **Barcode decoding of large sets of barcodes**

   a. **Precision and recall**

To evaluate the scalability of three barcode calling tools — QUIK, RandomBarcodes, and Columba — at large library sizes, we benchmarked each tool against simulated datasets of 1 and 4 million reads generated from barcode libraries of 170,000, 340,000, and 786,000 sequences (36 nt, medium error rate). For each combination, we measured the assignment rate, precision (fraction of assigned reads correctly called), recall (fraction of all reads correctly called), and runtime expressed as time per read (ms/read). All tools were executed under identical computational resources on the same cluster, using fixed threshold parameters per tool (QUIK: rejection threshold 8; RandomBarcodes: Nthresh 9; Columba: identity threshold 80).

Benchmarking across barcode libraries of 170K, 340K, and 786K sequences revealed consistent but tool-specific trends in precision, recall, and runtime as library size increased. **QUIK** maintained the highest recall across all conditions, ranging from 86.0 % at 170K to 82.5 % at 786K (1M reads), but showed the steepest decline in precision — from 99.2 % to 97.1 % — indicating that a larger search space leads to a progressively higher rate of ambiguous assignments at the fixed rejection threshold of 8. **RandomBarcodes** exhibited a more stable precision profile (99.6 % to 98.7 %), with recall remaining consistently around 55 % regardless of library size, suggesting that its conservative assignment strategy is relatively insensitive to library density. **Columba** showed the highest and most stable precision across all sizes (99.9 % to 99.5 %) and the most constant recall (~35 %), indicating that its identity-threshold-based alignment maintains strict specificity even as the library grows, at the cost of leaving the majority of reads unassigned. Results were highly reproducible between the 1M and 4M read datasets, confirming that the observed trends are not driven by sampling variation.

Figure 1. *Precision and recall of barcode-calling tools across increasing library sizes. Each panel shows results for one of three tools, plotted against barcode library size (170K, 340K, 786K sequences; 36 nt, medium error rate). The top row reports precision (percentage of assigned reads correctly identified) and the bottom row reports recall (percentage of total reads correctly assigned), for datasets of 1 million reads (left column) and 4 million reads (right column). All runs used tool-default thresholds (QUIK: rejection threshold 8; RandomBarcodes: nthresh 9; Columba: identity threshold 80)*

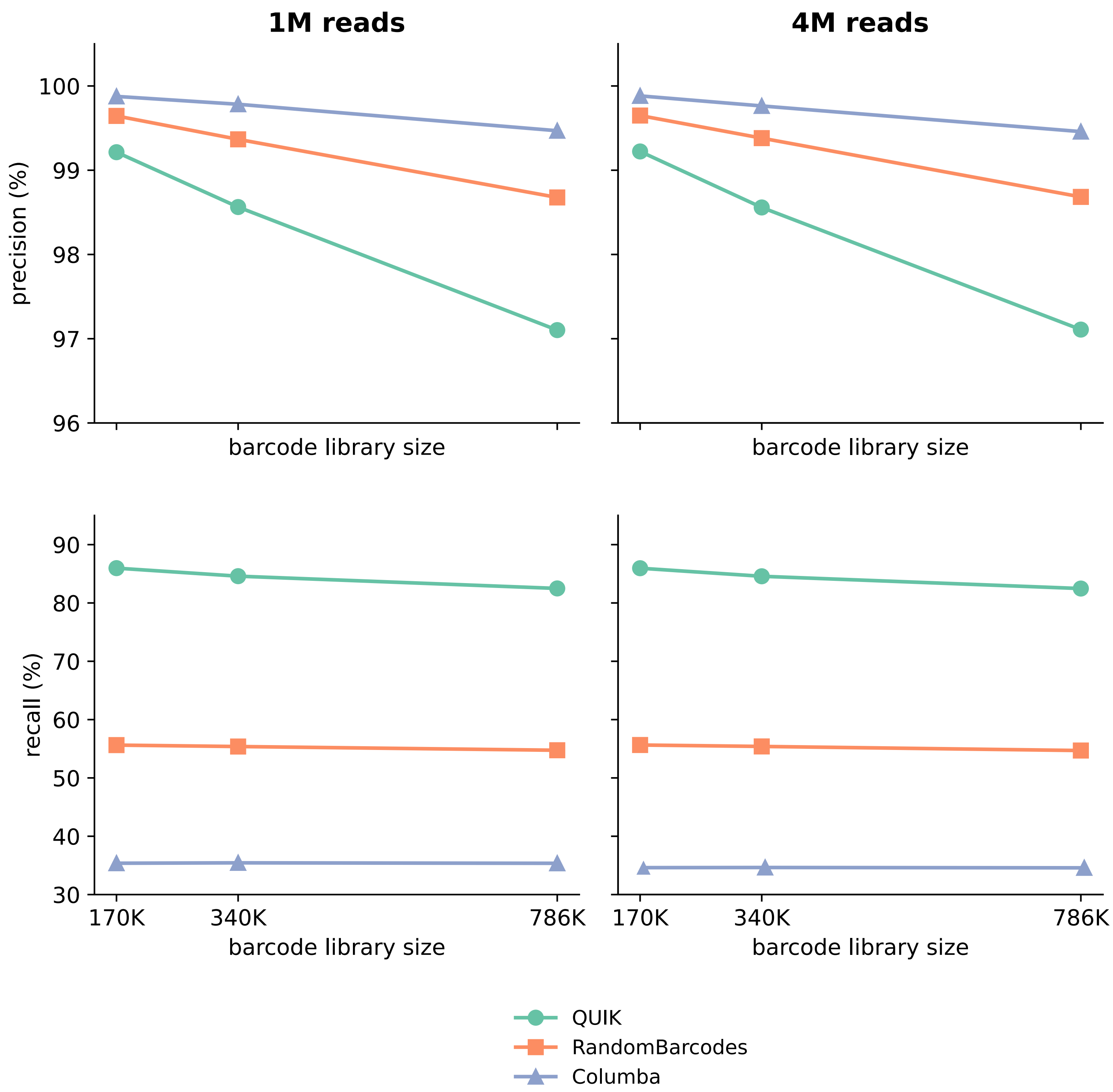

b. Runtime.

QUIK remained by far the fastest tool across all conditions, with a time per read of 0.073 ms at 170K scaling to 0.336 ms at 786K — a 4.6-fold increase that nonetheless kept per-read latency well below 0.5 ms. RandomBarcodes showed the most stable absolute runtime (9.10, 9.67, and 11.20 ms/read for 170K, 340K, and 786K, respectively), a 1.23-fold increase consistent with a near-linear dependence on library size. Columba, however, exhibited a non-linear runtime increase: while the step from 170K to 340K barcodes resulted in a modest 16% increase in per-read time (1.509 to 1.756 ms/read), the subsequent step to 786K barcodes produced a 2.37-fold increase relative to 340K (4.163 ms/read). Inspection of the Nextflow work directories revealed that this non-linearity affects both the index construction step and the alignment step. Index construction time scaled super-linearly from 21.8 s (170K) to 107.1 s (340K) to 710.5 s (786K), consistent with the O(n log n) complexity of BWT construction. The alignment throughput itself degraded from 633 reads/second at 170K to 544 reads/second at 340K and 229 reads/second at 786K. Resource traces confirmed that CPU utilization and memory pressure were comparable across all three runs (peak RSS 82–185 MB, approximately 79% CPU utilisation on all 16 threads), ruling out node-level contention or memory saturation as confounding factors. The most parsimonious explanation for the observed throughput drop is that the FM-index for 786K barcodes exceeds the processor's effective L3 cache capacity, increasing cache miss rates during the bidirectional BFM search that Columba performs per read. This type of non-linear degradation is a known characteristic of FM-index-based aligners operating on indices that cannot be held in fast memory, and should be considered when designing Columba-based workflows for barcode libraries in the high-hundreds-of-thousands range.

Figure 2. *Per-read runtime of barcode-calling tools across increasing library sizes. Time per read (milliseconds, log scale) is shown for the three tools as a function of barcode library size (170K, 340K, 786K sequences; 36 nt, medium error rate), for datasets of 1 million reads (left) and 4 million reads (right). Runtime was computed as total wall-clock processing time divided by the number of reads in the dataset.*

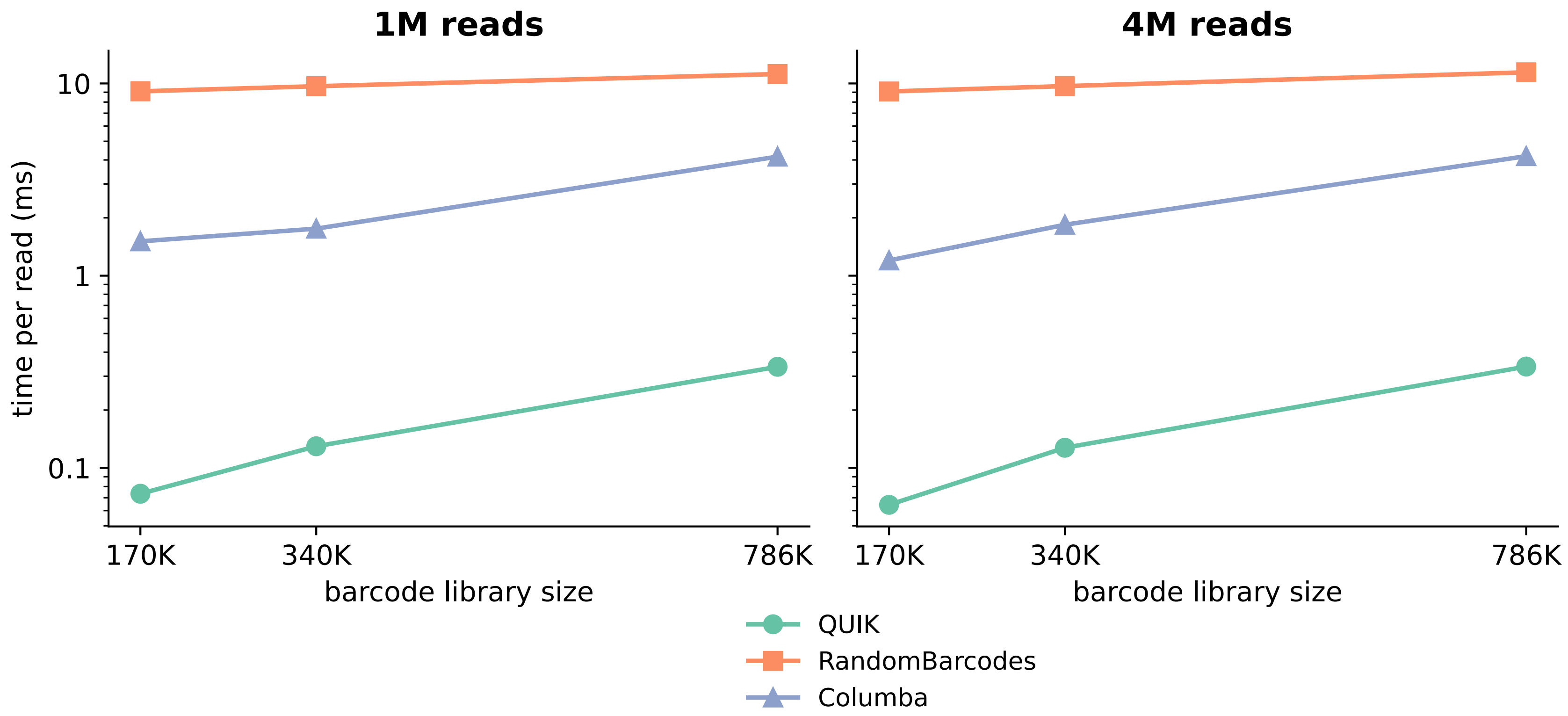

c. Comparison of QUIK strategies

Additionally, two QUIK decoding strategies, 4-mer (v4) and 4+7-mer (v1), were benchmarked across the three barcode library sizes (170K, 340K, and 786K) at sequencing depths of 1 million and 4 million reads. The 4-mer (v4) strategy was consistently faster, processing reads at 1.5–1.8× higher throughput than the 4+7-mer (v1) approach across all conditions (Figure 3 and Table 2). For the smallest library (170K barcodes), 4-mer (v4) achieved ~15,260 reads/s compared to ~8,430 reads/s for 4+7-mer (v1), corresponding to a 1.81× speed advantage. This difference was maintained at the 340K library size (7,840 vs. 4,270 reads/s; 1.84× faster), while it narrowed slightly at the largest library (786K barcodes; 2,910 vs. 1,861 reads/s; 1.56× faster). Throughput was stable between the 1 M and 4 M read datasets, confirming that the speed difference is an intrinsic property of the decoding strategy rather than a depth-dependent effect. Importantly, the gain in processing speed came at no cost to accuracy: assignment rate, precision, and recall were near-identical between strategies across all library sizes and read depths.

Figure 3. Computational speed of QUIK decoding strategies across barcode library sizes. *Throughput (reads per second, left) and time per read (milliseconds, right) of the two QUIK decoding strategies: 4-mer (v4, blue) and 4+7-mer (v1, orange), benchmarked on three barcode library sizes (170K, 340K, and 786K barcodes, all 36 nt). Solid bars represent results obtained with 1 million input reads; hatched bars represent 4 million input reads. The 4-mer (v4) strategy consistently achieves higher throughput and lower processing time per read compared to the combined 4+7-mer (v1) strategy, with the speed difference increasing with barcode library size.*

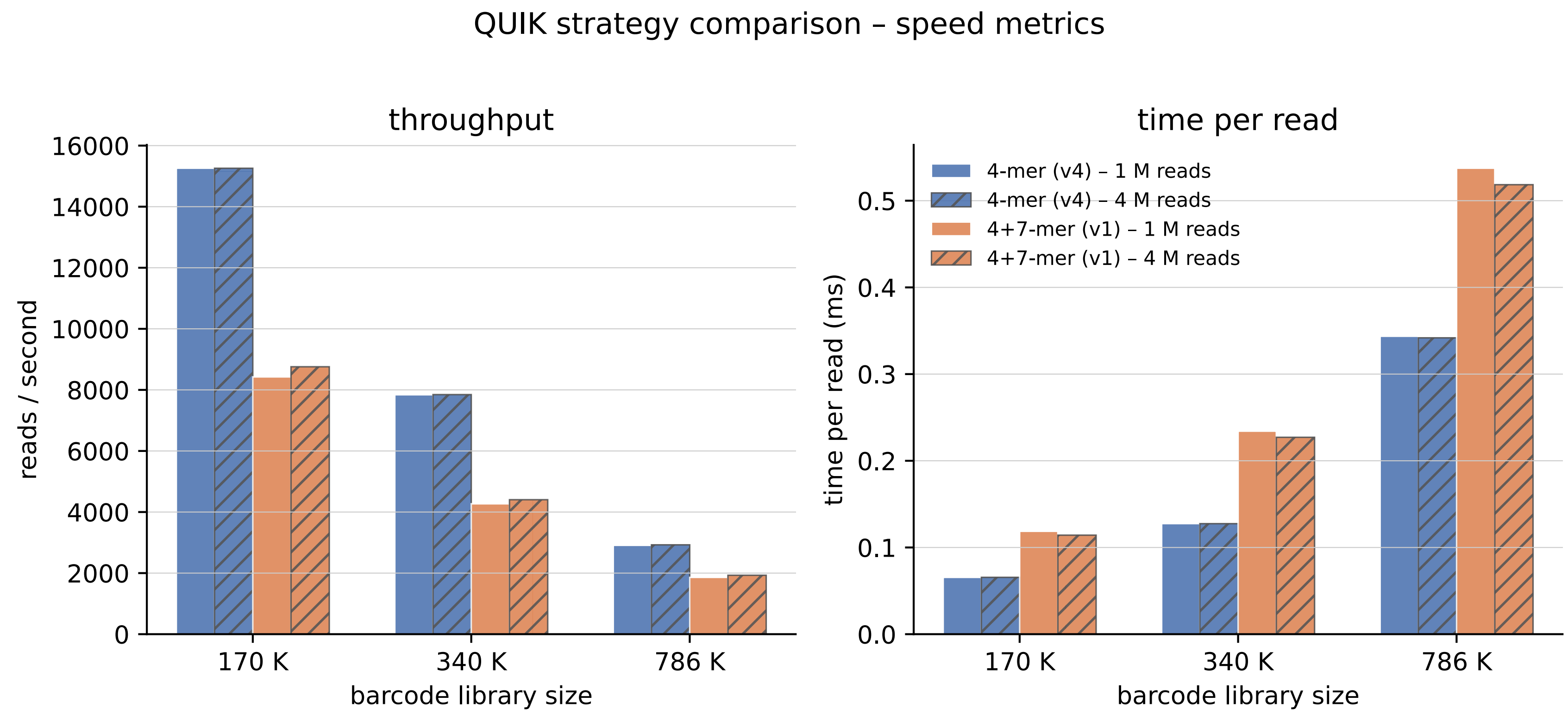

Table 1. Summary of results for simulated read assignments. This table evaluates the performance of QUIK against RandomBarcodes and Columba using three barcode sets (170K, 340K, 786K) and two scale-up scenarios (1M and 4M total reads). Metrics include total and correct assignments, assignment rate, precision, recall, and decoding speed (ms/read). Threshold values represent the optimal parameters used for each tool.

reads_label	tool	barcode_set	total_reads	assigned	correct	incorrect	assignment_rate	precision	recall	time_per_read_ms	threshold
**1M**	QUIK	170K_36nt	1000000	866505	859694	6811	86.6505	99.214	85.9694	0.073409	8.0
**1M**	QUIK	340K_36nt	1000000	858266	845933	12333	85.8266	98.563	84.5933	0.129651	8.0
**1M**	QUIK	786K_36nt	1000000	849508	824891	24617	84.9508	97.1022	82.4891	0.335898	8.0
**1M**	RandomBarcodes	170K_36nt	1000000	558270	556286	1984	55.827	99.6446	55.6286	9.09857	9.0
**1M**	RandomBarcodes	340K_36nt	1000000	557364	553835	3529	55.7364	99.3668	55.3835	9.67493	9.0
**1M**	RandomBarcodes	786K_36nt	1000000	554798	547457	7341	55.4798	98.6768	54.7457	11.1953	9.0
**1M**	Columba	170K_36nt	1000000	354182	353744	438	35.4182	99.8763	35.3744	1.509016	80.0
**1M**	Columba	340K_36nt	1000000	355187	354416	771	35.5187	99.7829	35.4416	1.756231	80.0
**1M**	Columba	786K_36nt	1000000	355511	353622	1889	35.5511	99.4687	35.3622	4.162556	80.0
**4M**	QUIK	170K_36nt	4000000	3465216	3438282	26934	86.6304	99.2227	85.957	0.064291	8.0
**4M**	QUIK	340K_36nt	4000000	3432511	3383015	49496	85.8128	98.558	84.5754	0.127347	8.0
**4M**	QUIK	786K_36nt	4000000	3397037	3298806	98231	84.9259	97.1083	82.4702	0.336743	8.0
**4M**	RandomBarcodes	170K_36nt	4000000	2233636	2225796	7840	55.8409	99.649	55.6449	9.08465	9.0
**4M**	RandomBarcodes	340K_36nt	4000000	2229752	2215950	13802	55.7438	99.381	55.3987	9.686725	9.0
**4M**	RandomBarcodes	786K_36nt	4000000	2217089	2187896	29193	55.4272	98.6833	54.6974	11.427575	9.0
**4M**	Columba	170K_36nt	4000000	1418051	1416383	1668	35.4513	99.8824	35.4096	1.20019	80.0
**4M**	Columba	340K_36nt	4000000	1420812	1417446	3366	35.5203	99.7631	35.4361	1.841099	80.0
**4M**	Columba	786K_36nt	4000000	1422888	1415182	7706	35.5722	99.4584	35.3796	4.186525	80.0

Table 2. Influence of k-mer strategy on QUIK decoding performance. Comparative results for 1M and 4M simulated read sets using 4-mer and 4+7-mer strategies. While both strategies maintain high precision (>97%), the 4-mer strategy exhibits significantly higher throughput (reads/sec) and lower latency (ms/read) across all barcode library sizes (170K–786K) compared to the 4+7-mer strategy.

reads_label	strategy	barcode_set	total_reads	total_runtime_s	reads_per_second	time_per_read_ms	assignment_rate	precision	recall	correct	incorrect	rejection_threshold
**1M**	4-mer (v4)	170K_36nt	1000000	65.5295	15260.3	0.0655295	86.6505	99.214	85.9694	859694	6811	8.0
**1M**	4-mer (v4)	340K_36nt	1000000	127.543	7840.5	0.127543	85.8266	98.563	84.5933	845933	12333	8.0
**1M**	4-mer (v4)	786K_36nt	1000000	343.652	2909.9	0.343652	84.9508	97.1022	82.4891	824891	24617	8.0
**1M**	4+7-mer (v1)	170K_36nt	1000000	118.683	8425.8	0.118683	86.6348	99.2146	85.9544	859544	6804	8.0

**1M**	4+7-mer (v1)	340K_36nt	1000000	234.194	4270.0	0.234194	85.8102	98.5619	84.5762	845762	12340	8.0
**1M**	4+7-mer (v1)	786K_36nt	1000000	537.486	1860.5	0.537486	84.9292	97.1052	82.4707	824707	24585	8.0
**4M**	4-mer (v4)	170K_36nt	4000000	262.154	15258.2	0.0655386	86.6304	99.2227	85.957	3438282	26934	8.0
**4M**	4-mer (v4)	340K_36nt	4000000	509.827	7845.8	0.127457	85.8128	98.558	84.5754	3383015	49496	8.0
**4M**	4-mer (v4)	786K_36nt	4000000	1367.26	2925.6	0.341816	84.9259	97.1083	82.4702	3298806	98231	8.0
**4M**	4+7-mer (v1)	170K_36nt	4000000	456.758	8757.4	0.114189	86.6141	99.223	85.9412	3437647	26918	8.0
**4M**	4+7-mer (v1)	340K_36nt	4000000	908.324	4403.7	0.227081	85.7943	98.5585	84.5576	3382305	49469	8.0
**4M**	4+7-mer (v1)	786K_36nt	4000000	2073.77	1928.9	0.518442	84.9052	97.1084	82.4501	3298004	98204	8.0
