# Supplementary material for: Benchmarking DNA barcode decoding strategies under high error rates
Source: BMC Bioinformatics. 2026 Jun 23;27:213. doi: 10.1186/s12859-026-06540-x (PMC13551773; doi:10.1186/s12859-026-06540-x)
Supplement: Supplementary file 2 [file 12859_2026_6540_MOESM2_ESM.pdf]

Additional file 2

1. Decoy sensitivity analysis

To assess the ability of barcode-calling tools to estimate precision in the absence of ground truth, a sensitivity analysis was performed usingdecoy barcodes. Decoy barcodes are sequences drawn from a second, non-overlapping barcode library that is disjoint from the library used to generate the sequenced reads. Because no read originates from a decoy barcode any assignment to a decoy sequence is, by definition, an erroneous call. This property allows precision to be estimated from the decoding output without requiring a ground truth answer file.

1.1 Experimental design

The real barcode set was supplemented with fraction of decoy barcodes (25 %, 50 %, 75 %, and 100 % of the real set size), and decoding was performed once against the enlarged combined reference. This approach was benchmarked using 200,000 simulated reads from an 85,000-barcode library (36n t) at a medium error rate, and evaluated across three tools: QUIK, RandomBarcodes, and Columba.

The decoy-based precision estimator was defined as:

$$precision\_decoy\_est = 1 - \frac{decoy_{assigned}}{assigned} \Big/ \frac{n\_decoy}{n\_total}$$

Where:

- *decoy $_{assigned}$ is the number of reads assigned to a decoy barcode*
- *assigned* represent the number of reads assigned to a barcode
- *n_decoy/n_total:* the fraction of decoy barcodes in the total barcode library

The normalization by the decoy library fraction (n_decoy / n_total) is essential because decoy barcodes act as a sampling probe of the full error space. An erroneous assignment is equally likely to land on any barcode in the reference; if decoys represent only 20 % of the combined library (as in the 25 % experiment, where 21,250 decoy sequences are added to 85,000 real ones), they will capture approximately 20 % of all errors, and the remaining 80 % will land on wrong real barcodes and be undetectable. Without normalization, the raw fraction of decoy-assigned reads systematically underestimates the total error rate by a factor equal to the decoy fraction, yielding inconsistent estimates across different decoy set sizes. Dividing by the decoy fraction scales the observed decoy error rate to the full library, producing a comparable estimate regardless of how many decoys were added.

1.2 Results

Across all three tools and all decoy fractions, the normalized estimator closely tracked the ground-truth precision. Using QUIK, the largest deviation between the decoy-based precision and true precision was 0.015 percentage points (at 25 % decoys), decreasing to 0.008 pp at higher fractions. RandomBarcodes and Columba showed similarly tight agreement. This confirms that the decoy-based estimator is a valid proxy for true precision and can be applied in real experiments where ground truth is unavailable (see Additional Table 1).

A secondary observation is that precision decreases modestly but consistently as the decoy fraction increases — for QUIK, from 99.59 % (no decoys) to 99.25 % (100 % decoys). This reflects the enlarged search space: with more candidate barcodes in the reference, borderline reads have a higher probability of being assigned to an incorrect sequence. This effect is a genuine property of the tool under realistic library conditions, not an artifact of the analysis.

Additional Table 1. Decoy-sensitivity analysis. The table shows the number of total and correct assignments, as well as the incorrect assignments to real and decoy barcodes. Column precision_true shows the true precision that can be computed as we know the ground truth. Column precision_decoy_est shows the decoy-based precision, which represents the precison that one can compute in real experiments where the ground truth is unknown. Columns n_decoy_barcodes and decoy_level show the number of decoy barcodes added to the real barcode libraries and the fraction it represents from the total 85,000 real barcodes.

tool	decoy_level	n_decoy_barcodes	total_reads	assigned	unassigned	correct	incorrect_real	decoy_assigned	assignment_rate	precision_true	precision_decoy_est	recall
quik	noDecoy	0	200000	174967	25033	174249	718	0	87.4835	99.5896		87.12
quik	25pct	21250	200000	174508	25492	173627	710	171	87.2540	99.4952	99.5101	86.81
quik	50pct	42500	200000	174138	25862	173104	694	340	87.0690	99.4062	99.4143	86.55
quik	75pct	63750	200000	173798	26202	172616	677	505	86.8990	99.3199	99.3220	86.31
quik	100pct	85000	200000	173473	26527	172164	661	648	86.7365	99.2454	99.2529	86.08
randombarcodes	noDecoy	0	200000	112252	87748	112065	187	0	56.1260	99.8334		56.03
randombarcodes	25pct	21250	200000	112140	87860	111911	189	40	56.0700	99.7958	99.8217	55.96
randombarcodes	50pct	42500	200000	112122	87878	111848	187	87	56.0610	99.7556	99.7672	55.95
randombarcodes	75pct	63750	200000	112067	87933	111768	178	121	56.0335	99.7332	99.7481	55.88
randombarcodes	100pct	85000	200000	111953	88047	111606	173	174	55.9765	99.6900	99.6892	55.80

**columba**	noDecoy	0	200000	70775	129225	70733	42	0	35.3875	99.9407		35.37
**columba**	25pct	21250	200000	70781	129219	70733	42	6	35.3905	99.9322	99.9576	35.37
**columba**	50pct	42500	200000	70790	129210	70733	42	15	35.3950	99.9195	99.9364	35.37
**columba**	75pct	63750	200000	70800	129200	70733	42	25	35.4000	99.9054	99.9176	35.37
**columba**	100pct	85000	200000	70805	129195	70729	42	34	35.4025	99.8927	99.9040	35.37

1.3 Recommendation

The estimator performed well even at a 25 % decoy fraction, with deviations from ground truth below 0.02 percentage points for all three tools. However, lower decoy fractions yield fewer absolute decoy assignments (e.g. 171 decoy assignments for QUIK at 25 %), which increases the variance of the estimate in datasets with fewer reads. A decoy fraction of 25–50 % of the real barcode set size represents a practical optimum: it provides a sufficient number of decoy assignments for a precision estimate while minimizing the enlargement of the reference library and the associated reduction in assignment rate precision. Fractions above 50 % offer diminishing returns in estimation of accuracy and impose a progressively competitive burden on the tool.
