# Supplementary material for: Benchmarking DNA barcode decoding strategies under high error rates
Source: BMC Bioinformatics. 2026 Jun 23;27:213. doi: 10.1186/s12859-026-06540-x (PMC13551773; doi:10.1186/s12859-026-06540-x)

Additional file 3

To characterize the separability of the barcode libraries used in the benchmarks, we computed three distance metrics for each library: the nearest-neighbour (NN Hamming distance (the Hamming distance from each barcode to its closest neighbour in the set a sampled pairwise Hamming distance (from a random sample of barcode pairs), and NN Levenshtein distance (computed on a subsample of 3000 barcodes). Analysis performed for all six library configurations evaluated: 21K, 42K, and 85K barcodes with lengths of 28 nt and 36 nt.

For the 28 nt libraries, the median NN Hamming distance was 11 (21K), 10 (42K), and 10 (85K), with minimum values of 6, 6, and 5, respectively (Fig. 1). For the 36 nt libraries, NN Hamming distance was 16 (21K), 15 (42K), and 15 (85K), with minimum values of 10, 10 and 9 (Fig. 2). In both cases, the median NN Hamming distance corresponds to 38–40 % of barcode length, indicating that all libraries maintain substantial sequence separation across the full range of library sizes tested. The pairwise Hamming distributions were broader and shifted to higher values (median 21 for 28 nt and 27 for 36 nt across all library sizes reflecting that any two randomly chosen barcodes are on average much more than a barcode and its nearest neighbour. NN Levenshtein distances followed the same Hamming distances but were consistently lower by approximately one to two units (median 10 for 28 nt and 14 for 36 nt), as expected given that Levenshtein distance allows insertions and deletions in addition to substitutions and is therefore a more permissive measure.

A slight reduction in minimum NN distances was observed as library size from 21K to 85K, for example, the NN Hamming minimum decreased from 6 to 5 for the 28 nt set from 10 to 9 for the 36 nt set, consistent with the progressive filling of the sequence space at larger library sizes. Overall, all six libraries maintained minimum NN distances well above the error thresholds used by the evaluated tools (typically 8–9 substitutions) supporting reliable barcode decoding across the evaluated experimental conditions.

Table 1. Summary statistics of sequence distance distributions for the barcode libraries used in the benchmark. For each of the six barcode libraries (three sizes — 21K, 42K, and 85K sequences — at two barcode lengths — 28 nt and 36 nt), three distance metrics are reported. The nearest-neighbour (NN) Hamming distance was computed exhaustively for every barcode against all other barcodes in the library; the minimum, first quartile (Q1), median, third quartile (Q3), maximum, and mean are reported. The pairwise Hamming distance was estimated from 200,000 randomly sampled barcode pairs; only the minimum, median, and maximum are shown to summarise the distribution shape. The nearest-neighbour Levenshtein distance was computed on a random sample of 3000 barcodes drawn from each library; the minimum, Q1, median, and maximum are reported. Levenshtein distance allows insertions and deletions in addition to substitutions and therefore yields systematically lower values than the corresponding Hamming distance.

library	n_bar	bar_length	ham_nn min	ham_nn q1	ham_nn median	ham_nn q3	ham_nn max	ham_nn mean	ham_pair min	ham_pair median	ham_pair max	lev_nn min	lev_nn q1	lev_nn median	lev_nn max
**21K_28nt**	21000	28	6.0	10.0	11.0	11.0	13.0	10.67	10.0	21.0	28.0	7.0	9.0	10.0	11.0
**21K_36nt**	21000	36	10.0	15.0	16.0	16.0	18.0	15.40	15.0	27.0	36.0	10.0	13.0	14.0	15.0
**42K_28nt**	42000	28	6.0	10.0	10.0	11.0	12.0	10.26	9.0	21.0	28.0	7.0	9.0	10.0	11.0
**42K_36nt**	42000	36	10.0	14.0	15.0	16.0	17.0	14.90	13.0	27.0	36.0	10.0	13.0	14.0	15.0
**85K_28nt**	85000	28	5.0	9.0	10.0	10.0	12.0	9.84	9.0	21.0	28.0	6.0	9.0	10.0	11.0
**85K_36nt**	85000	36	9.0	14.0	15.0	15.0	17.0	14.43	14.0	27.0	36.0	11.0	13.0	14.0	15.0

Figure 1. Sequence distance distributions of the 28 nt barcode libraries used in the benchmark. Each row corresponds to a barcode library of increasing size: 21K (top), 42K (middle), and 85K (bottom) sequences. Columns show three complementary distance metrics. Left column: Nearest-neighbour (NN) Hamming. Middle column: Pairwise Hamming distance. Right column: Nearest-neighbour Levenshtein distance. The red dashed line marks the minimum of the distribution for the two NN panels, and the median for the pairwise Hamming panel.

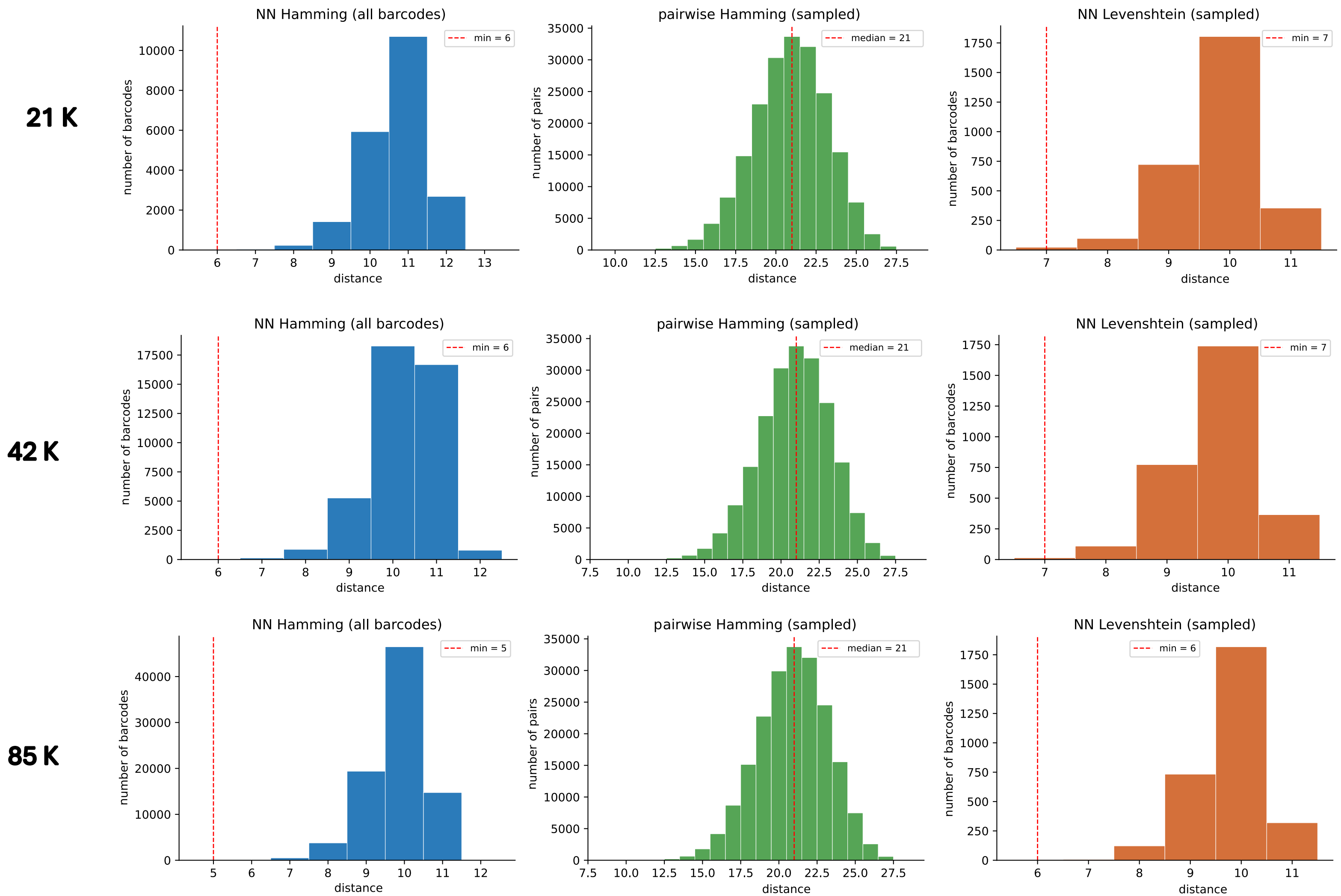

Figure 2. Sequence distance distributions of the 36 nt barcode libraries used in the benchmark. Each row corresponds to a barcode library of increasing size: 21K (top), 42K (middle), and 85K (bottom) sequences. Columns show three complementary distance metrics. Left column: Nearest-neighbour (NN) Hamming. Middle column: Pairwise Hamming distance. Right column: Nearest-neighbour Levenshtein distance. The red dashed line marks the minimum of the distribution for the two NN panels, and the median for the pairwise Hamming panel.

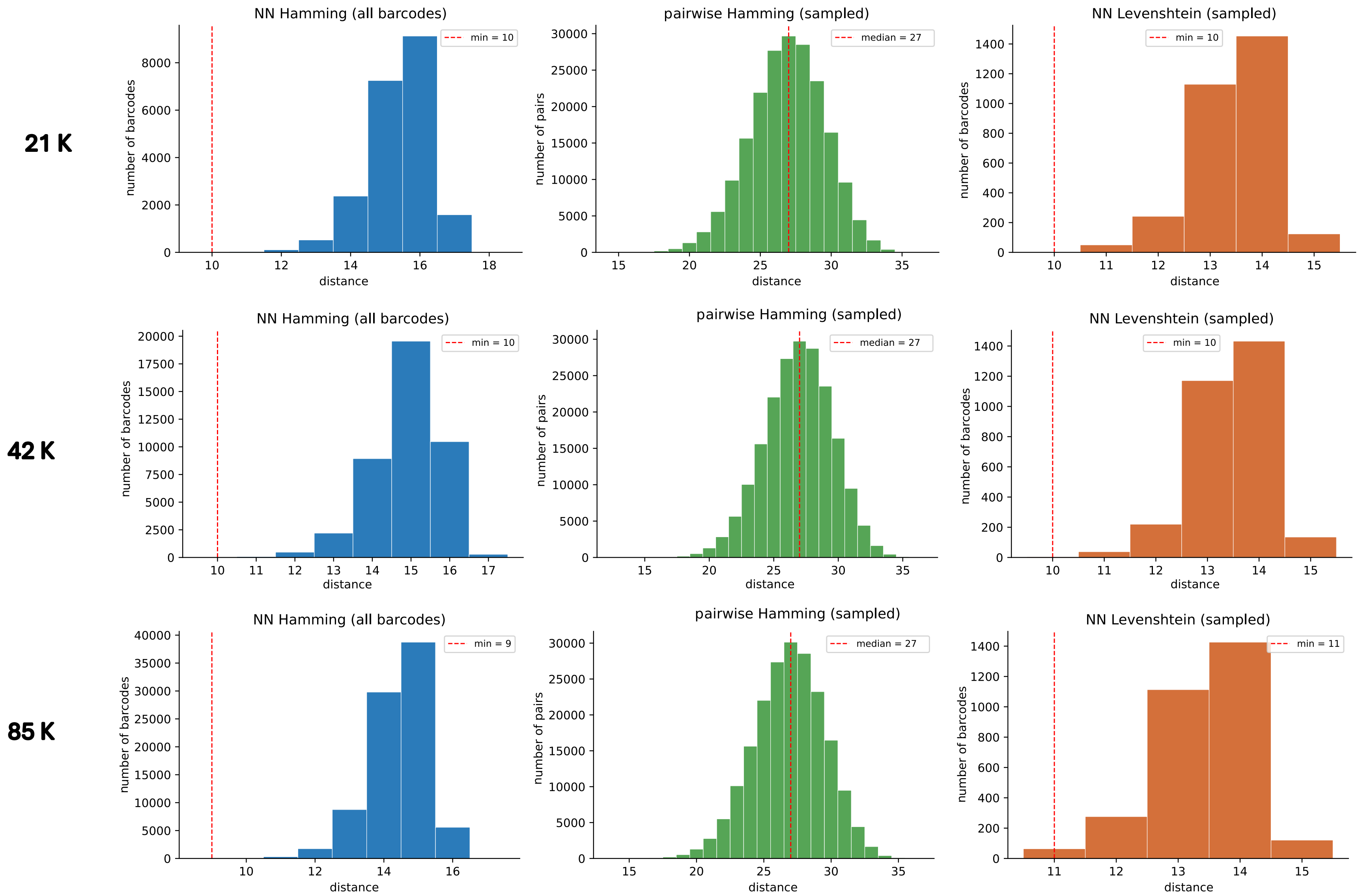

Supplement: Supplementary file 3 [file 12859_2026_6540_MOESM3_ESM.pdf]
